# Imaging multiple sclerosis pathology at 160 μm isotropic resolution by human whole-brain ex vivo magnetic resonance imaging at 3 T

**DOI:** 10.1038/s41598-021-94891-1

**Published:** 2021-07-29

**Authors:** Matthias Weigel, Peter Dechent, Riccardo Galbusera, Erik Bahn, Govind Nair, Po-Jui Lu, Ludwig Kappos, Wolfgang Brück, Christine Stadelmann, Cristina Granziera

**Affiliations:** 1 Translational Imaging in Neurology (ThINk) Basel, Department of Biomedical Engineering, University Hospital Basel and University of Basel, Gewerbestrasse 14, 4123 Allschwil, Switzerland; 2 Neurologic Clinic and Policlinic, Departments of Medicine, Clinical Research and Biomedical Engineering, University Hospital Basel and University of Basel, Petersgraben 4, 4031 Basel, Switzerland; 3 Research Center for Clinical Neuroimmunology and Neuroscience Basel (RC2NB), University Hospital Basel and University of Basel, Petersgraben 4, 4031 Basel, Switzerland; 4 Division of Radiological Physics, Department of Radiology, University Hospital Basel, Petersgraben 4, 4031 Basel, Switzerland; 5 Department of Cognitive Neurology, MR-Research in Neurosciences, University Medical Center Göttingen, Robert-Koch-Strasse 40, 37075 Göttingen, Germany; 6 Institute of Neuropathology, University Medical Center Göttingen, Robert-Koch-Strasse 40, 37075 Göttingen, Germany; 7 Quantitative MRI Core Facility, National Institute of Neurological Disorders and Stroke, National Institutes of Health, 10 center drive, Bethesda, MD USA

**Keywords:** Neuroscience, Brain, Multiple sclerosis, Biomedical engineering, Imaging techniques

## Abstract

Postmortem magnetic resonance imaging (MRI) of the fixed healthy and diseased human brain facilitates spatial resolutions and image quality that is not achievable with in vivo MRI scans. Though challenging—and almost exclusively performed at 7 T field strength—depicting the tissue architecture of the entire brain in fine detail is invaluable since it enables the study of neuroanatomy and uncovers important pathological features in neurological disorders. The objectives of the present work were (1) to develop a 3D isotropic ultra-high-resolution imaging approach for human whole-brain ex vivo acquisitions working on a standard clinical 3 T MRI system; and (2) to explore the sensitivity and specificity of this concept for specific pathoanatomical features of multiple sclerosis. The reconstructed images demonstrate unprecedented resolution and soft tissue contrast of the diseased human brain at 3 T, thus allowing visualization of sub-millimetric lesions in the different cortical layers and in the cerebellar cortex, as well as unique cortical lesion characteristics such as the presence of incomplete/complete iron rims, and patterns of iron accumulation. Further details such as the subpial molecular layer, the line of Gennari, and some intrathalamic nuclei are also well distinguishable.

## Introduction

Postmortem magnetic resonance (MR) imaging of the formalin-fixed healthy and diseased human brain offers the possibility to gain a deep understanding of neuromorphology and neuropathology, including the option to compare macro- and microanatomical and tissue-related features to histopathology^1–3^. Indeed, postmortem imaging benefits from a number of advantages compared to in vivo imaging such as the lack of many sources of artifacts and the opportunity to perform long scan sessions (i.e., ranging from hours to days), which enable spatial resolutions and image quality not achievable with in vivo scans^2,4–6^. Postmortem imaging of fixed brains, however, is also characterized by several challenges, encompassing the changes in MRI properties due to fixation, large image artifacts resulting from the magnetic susceptibility differences that occur at (residual) air—tissue boundaries, etc.^1,7–9^.

Yet, postmortem whole brain imaging is important to observe neuroanatomic relationships across distant brain regions, to provide neuroanatomical reference points in standard stereotactic space and to understand neurological diseases affecting the entire brain structure.

In contrast to the imaging of tissue-blocks or slices^10–12^—which is usually performed in small-bore scanners and/or using specialized receiver-coils—ex vivo whole-brain MR imaging has been frequently performed at 7 T main field strength^4,13^, since it generally provides a higher signal-to-noise-ratio (SNR) per unit time. SNR is usually the most limiting factor in terms of achievable resolution; and increasing SNR solely by signal averaging leads to an unfavorable quadratic increase of acquisition time. In contrast, 3 T MR systems have the benefit of being much more widely available, are more economic in operation, provide quite homogeneous excitation as well as reception fields and *B*_0_ dependent physical effects like susceptibility are expected to be more comparable to the in vivo condition from clinical routine at 3 T.

The purpose of this work was to develop—and to test for the viable boundaries of—an ultra-high-resolution MR acquisition for human whole-brain ex vivo imaging with strong soft tissue contrast on a standard clinical 3 T MR system. For this purpose, we studied the entire brain of two multiple sclerosis (MS) patients and explored the sensitivity and specificity of the acquired ultra-high-resolution images to pathological features, both in white matter and in the cortex. Five different base protocols were established to acquire isotropic 3D resolutions between 160 and 270 µm within a few hours up to a few days. The resulting MR images display unprecedented quality and resolution of the diseased human brain at 3 T field strength, opening therefore new perspectives for the investigation of sub-millimetric MS pathology in broadly available scanner types, without the need of ad-hoc customized head-coils, offline reconstruction and ultra-high field scanners. Moreover, images at such a spatial resolution and contrast may be also exploited for neuroanatomical and neuropathological educational purposes, where availability of brain tissue and pathological tissue is limited.

## Materials and methods

### Specimen preparation and experimental setup

The following experiments were approved by the ethical review committee of the Medical Center of Göttingen (29/9/10). All methods were carried out in accordance with the relevant guidelines and regulations. In further detail, clinical data and autopsy brain tissue were collected following established standard operating procedures (SOPs) developed within the MS Brain Bank of the KKNMS (German competence network for multiple sclerosis). The participants provided written informed consent for brain autopsy and for donation of their brain for research.

The whole brain of two deceased patients diagnosed with multiple sclerosis were investigated. The first was a 58-year-old man with a 24-year history of disease and the second a 66-year-old man with an unknown disease time. The autopsy had been performed 24 h postmortem and the brains were fixated directly in 4% neutral buffered formaldehyde solution (formalin) for 8 and 12 months, respectively.

To position the brains within the scanner, we used a 3D printed cylindric brain container that was shaped to perfectly fit the 20ch head coil^3,14,15^. A special circular surface was also fixed within the cylinder to keep the brain stable and avoid movements. Within the MRI-compatible container the brain was immersed in Fomblin^®^ perfluoropolyether (Solvay Specialty Polymers USA, LLC, West Deptford, NJ, USA). This fluoropolymer has a magnetic susceptibility similar to that of tissue and lacks any signal in hydrogen-based MRI^16^. Since air bubbles produce substantial susceptibility artifacts, these were removed through the degassing ports of the container using a vacuum pump^3,14,15^.

All experiments were performed on a 3 T whole-body MR system (Prisma^Fit^, Siemens Healthineers, Erlangen, Germany) with a maximal gradient amplitude of 80 mT/m and a maximal slew rate of 200 mT/m/ms. For radiofrequency (RF) transmission the built-in body coil was employed. For RF reception the standard, manufacturer-supplied 20-channel phased-array head and neck coil was used.

The overall protocol consisted of initial localizer scans for controlling the position and alignment of the brain and its container both in the RF head and neck coil and in the scanner isocenter. Subsequently, a series of acquisitions with one of the here suggested ultra-high-resolution MR imaging (URI) acquisition protocols was performed. For these acquisitions the advanced shim routine provided by the manufacturer was turned on. The maximum number of protocol repetitions was defined by the whole time that was available in our respective measurement slot. The shortest measurement slot had ~ 7 h, the longest had ~ 90 h (cf. Supplementary Tab. S1).

After completion from all acquisitions in the different measurement slots a 3D-printed individualized cutting box based on the 3D-information contained in the MRI images was designed^3,14^. This procedure ensures a good pairing between histology and imaging. Subsequently, the match between the digitized brain slabs surfaces and the corresponding MR images was further refined by manual image registration. Afterwards, carefully selected regions of interest (ROI) at MRI were cut and stained with Luxol fast blue (LFB, for myelin) and immunohistochemically with Myelin Basic Protein (MBP, for myelin) and MHC II (for microglia/macrophages).

### The 3 T URI acquisition approach

Five URI base protocols with isotropic 3D resolutions of 160 μm, 180 μm, 200 μm, 240 μm, and 270 μm were established. The protocols are based on the principle of a common and basic RF spoiled gradient echo sequence, often referred to as a fast low-angle shot (FLASH) sequence^17^. Since vendor MRI sequences are typically restricted regarding what protocol parameters can be modified and which parameter range is permitted, an in-house developed FLASH sequence with a simple linear 3D phase encoding loop structure was employed. As an additional benefit, with the in-house sequence a more precise monitoring of the needed MR system performance was feasible for test purposes in the beginning.

All five base protocols used a constant echo time TE = 18 ms, a constant receiver bandwidth of 50 Hz/Px, and transversal slice orientation (no angulation). This overall procedure ensured images of similar contrast and imaging behavior. The resolution-dependent parameters were: (1) isotropic 3D resolution (160 μm)^3^, field-of-view (FOV) = 192 × 150 cm^2^, matrix = 1200 × 936, slice thickness = 0.16 mm, slices per slab = 704, phase encoding direction right to left, flip angle = 31 deg, TR = 38 ms, TA_base_ = 06:57:20 h; (2) isotropic 3D resolution (180 μm)^3^, FOV = 192 × 161 cm^2^, matrix = 1072 × 896, slice thickness = 0.18 mm, slices per slab = 768, phase encoding direction right to left, slice oversampling = 80%, flip angle = 31 deg, TR = 37 ms, TA_base_ = 07:04:49 h; (3) isotropic 3D resolution (200 μm)^3^, FOV = 192 × 156 cm^2^, matrix = 960 × 780, slice thickness = 0.20 mm, slices per slab = 768, phase encoding direction right to left, slice oversampling = 33.3%, flip angle = 31 deg, TR = 37 ms, TA_base_ = 08:12:33 h; (4) isotropic 3D resolution (240 μm)^3^, FOV = 192 × 190 cm^2^, matrix = 800 × 792, slice thickness = 0.24 mm, slices per slab = 640, phase encoding direction right to left, slice oversampling = 80%, flip angle = 30 deg, TR = 36 ms, TA_base_ = 09:08:07 h; (5) isotropic 3D resolution (270 μm)^3^, FOV = 192 × 192 cm^2^, matrix = 704 × 704, slice thickness = 0.27 mm, slices per slab = 576, phase encoding direction right to left, slice oversampling = 77.8%, flip angle = 30 deg, TR = 35 ms, TA_base_ = 07:00:31 h. Supplementary Table S1 summarizes the corresponding acquisition times again and also informs about repeated acquisitions for later averaging.

### Elective postprocessing of image data

For image reconstruction, the standard postprocessing pipeline within the MR system was used, consisting of the basic Fast Fourier Transform (FFT) and the standard intensity normalization filter (vendor name “prescan normalize”). Neither other kinds of filtering nor interpolation algorithms were utilized. The images were exported into the standard DICOM format.

The repeated volume acquisitions of identical resolution were manually averaged up slice by slice directly on the MR system and were then also exported as DICOM images. No kinds of image registration procedures were performed for this procedure.

The presented full-size and zoomed MR images in all figures were generated with the free software ITK-SNAP 3.6.0^18^.

### Expert reading

Recommendations for the minimum number of averages (avg_min-SNR_, Supplementary Tab. S1) that should be performed for a given resolution are based on the expertise of seven MRI experienced clinicians and scientists (experience for 12.1 ± 7.8 years, mean ± standard deviation). All of them took part in a small survey by evaluating respective series of images with increasing averages, judging about the displayed SNR and overall image quality.

## Results

Based on representative imaging examples in transverse, sagittal, and coronal orientation, Figs. 1, 2, 3, 4, 5, 6, 7, 8, 9 and 10 as well as Supplementary Figs. S1 to S6 depict the characteristics of the suggested ex vivo 3 T URI-FLASH approach. As it can be deduced from all the figures, the complete setup of fixed brain, dedicated container, hardware, and MRI sequence is stable enough for measuring isotropic ultra-high resolutions up to 160 μm in 3D with a pronounced tissue contrast over more than 90 h (cf. Supplementary Tab. S1).

**Figure 1:**
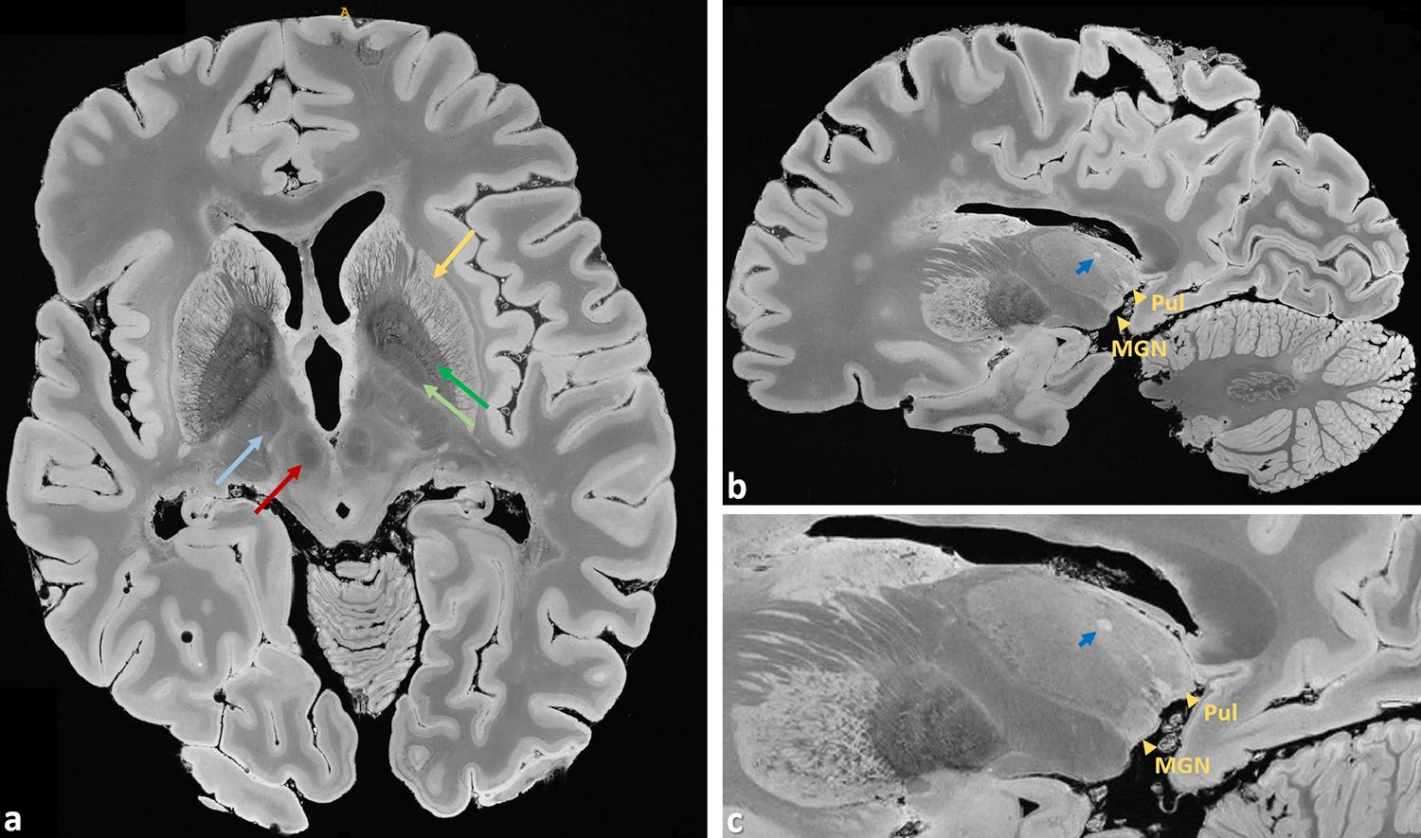
Brain #1, representative images from the 200 µm acquisition demonstrate the richness of anatomical details that can be observed in the URI-FLASH images. They display a combination of resolving capability and soft tissue contrast that can just not be obtained with clinical 3 T imaging. (**a**) Originally acquired transverse slice depicting, e.g., the putamen (gold arrow), external globus pallidus (dark green arrow), internal globus pallidus (light green arrow), red nucleus (red arrow), and subthalamic nucleus (light blue arrow). (**b**) Sagittal reformation and (**c**) enlargement of the thalamus and the basal ganglia. Gold arrowheads mark the thalamic nuclei medial geniculate nucleus (*Med Gen*) and pulvinar (*Pul*). A thalamic lesion can also be observed (dark blue arrow).

**Figure 2:**
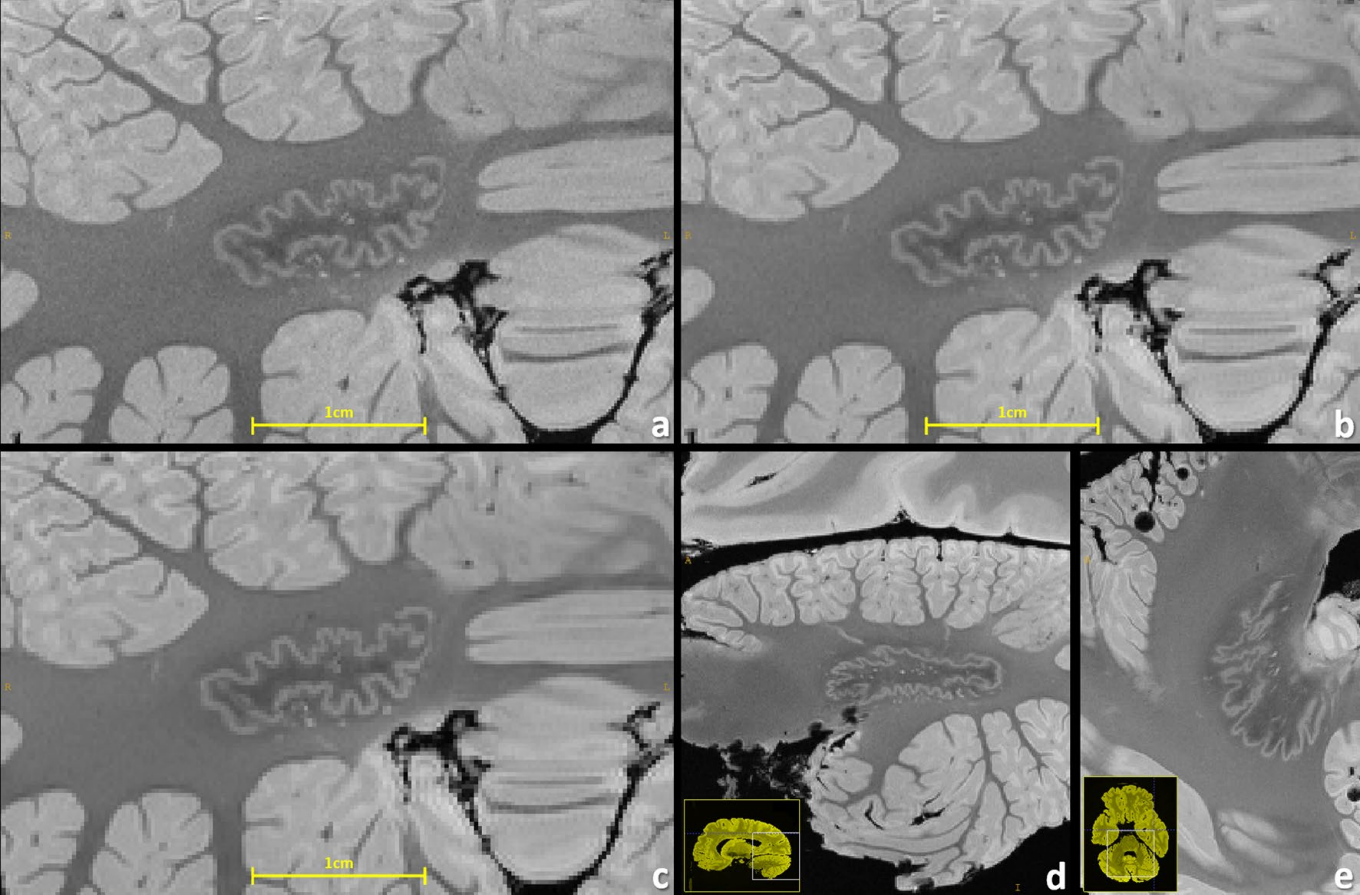
Brain #1, comparison of resolving power visualized at the cerebellar dentate nucleus with 160 µm (**a**), 200 µm (**b**), and 240 µm (**c**) resolution. Particularly for the 240 µm resolution the structures get pixelated, demonstrating that there is still gain in increasing even such ultra-high resolutions for brain MRI. A sagittal reformation (**d**) and a transverse slice (**e**) of the dentate nucleus are also depicted (200 µm resolution).

**Figure 3:**
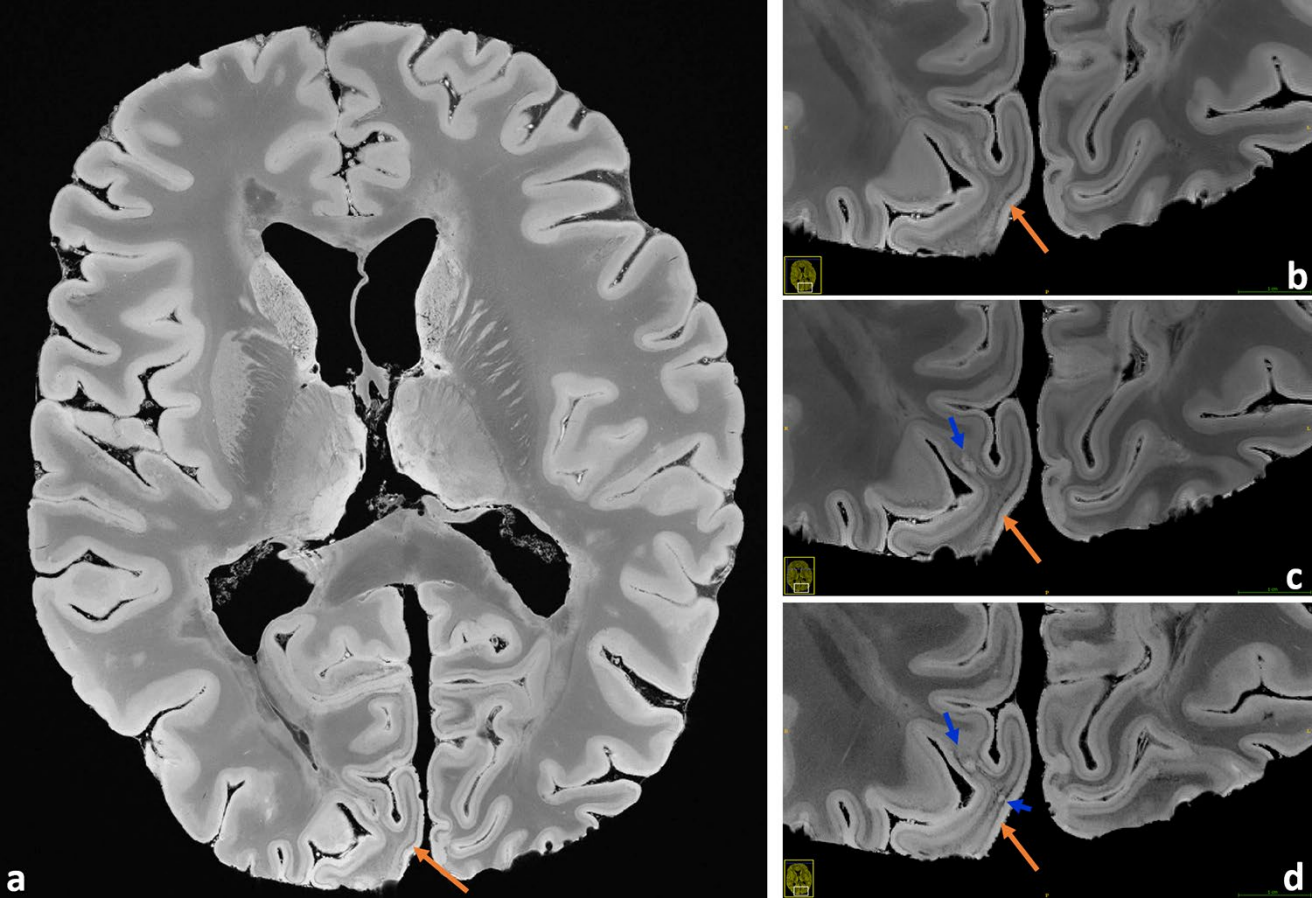
Very good observation of the line of Gennari in the occipital lobe (brain #1, orange arrows). (**a**) Transverse overview slice, also underlining the pronounced depiction of soft tissue structures; magnifications for the spatial resolutions 240 μm (**b**), 200 μm (**c**), and 160 μm (**d**) are contrasted. Some small lesions are better delineated at the highest resolution (blue arrows).

**Figure 4:**
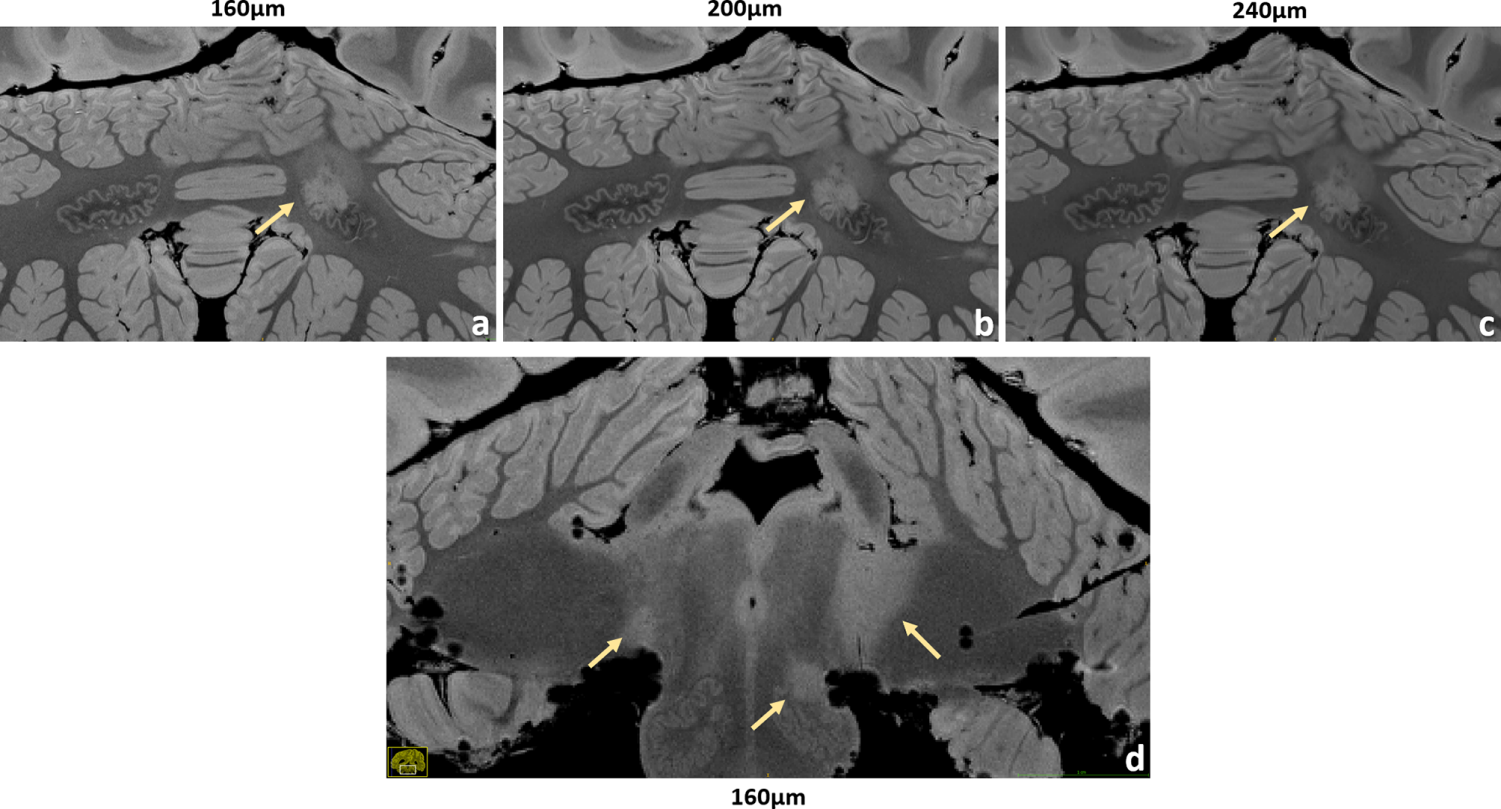
Brain #1, examples of infratentorial lesions. A dentate nucleus lesion is contrasted at the 160 µm (**a**), 200 µm (**b**) and 240 µm (**c**) resolution. The anatomical features of this nucleus are depicted in great detail, especially at larger magnification. In this case, the left dentate nucleus is lesioned in its rostral part. A very detailed anatomical representation of brainstem and cerebellum is shown at 160 µm resolution (**d**). A small lesion of the left inferior olivary nucleus is well visible and clearly defined. Moreover, typical bilateral MS lesions at the cerebellopontine angle can be observed here, more extended on the left side.

**Figure 5:**
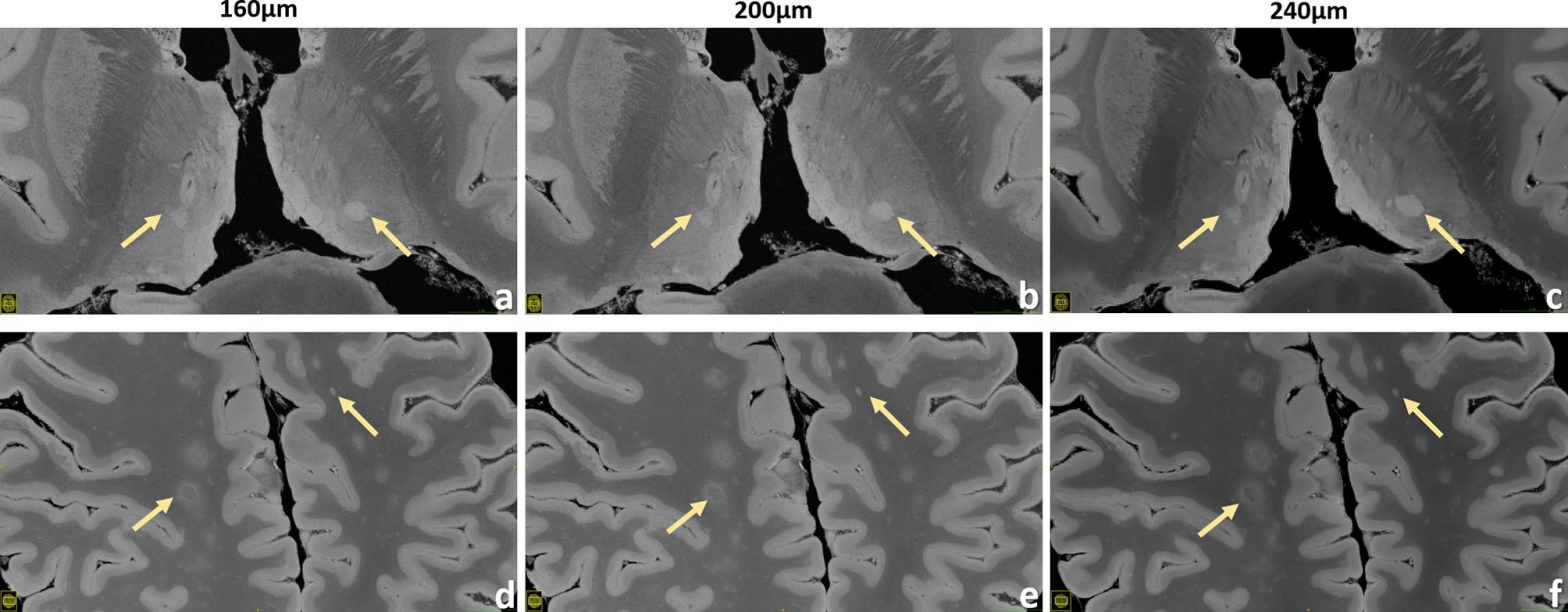
Brain #1, examples of thalamic lesions (upper row) and some exemplary subcortical WM lesions (*lower row*) are demonstrated for the 160 µm (**a**,**d**), 200 µm (**b**,**e**) and 240 µm (**c**,**f**) resolution.

**Figure 6:**
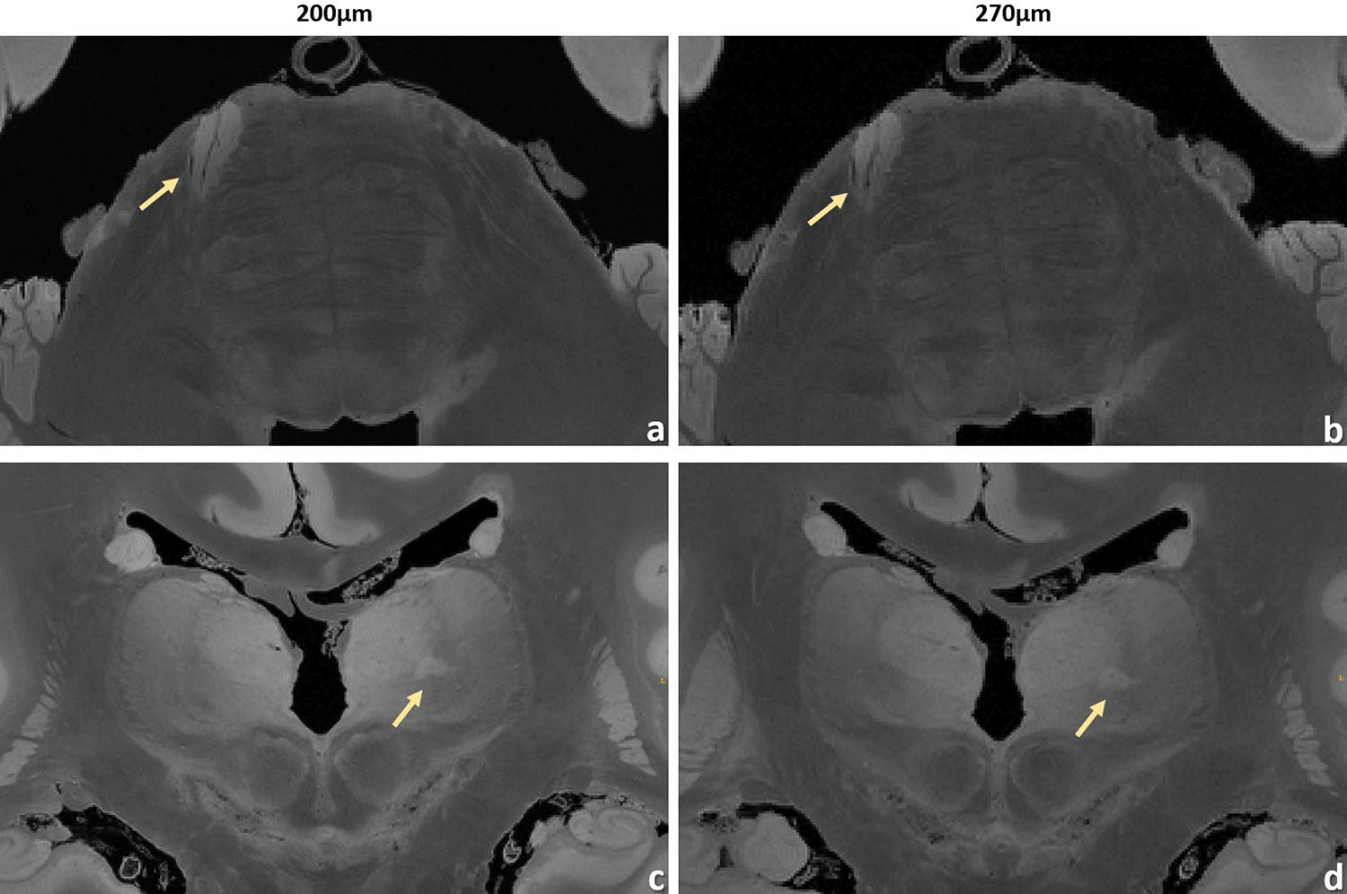
URI-FLASH measurements in MS brain #2. Upper row*:* zoomed views of an axial section with 200 µm resolution (**a**) and with the “fast acquired” 270 µm resolution (**b**), showing a pontine lesion. The central vein is clearly recognizable. Lower row: zoomed views of a coronal section with 200 µm resolution (**c**) and with 270 µm resolution (**d**), showing a thalamic lesion.

**Figure 7:**
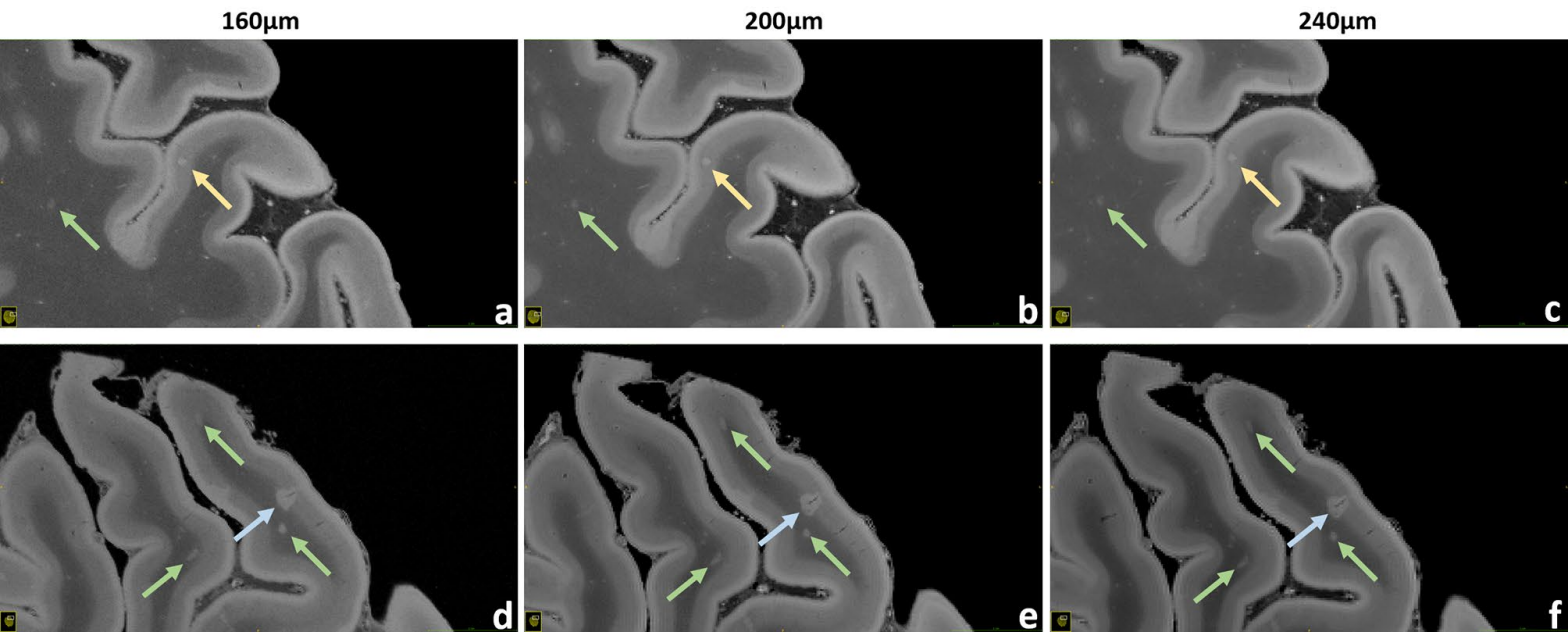
Brain #1, exemplary intracortical, leukocortical, and WM lesions. Upper row: a pure intracortical lesion acquired with 160 µm (**a**), 200 µm (**b**) and 240 µm (**c**) resolution is shown (gold arrows). This very small round hyperintense area is placed in the middle of the cortex, without touching the WM or the subpial surface. Lower ro*w*: a leukocortical lesion acquired with 160 µm (**d**), 200 µm (**e**) and 240 µm (**f**) resolution is depicted (blue arrows). In this case the hyperintense area is located across the border between WM and GM. A thin linear hypointensity, representing the “central vein”, is well visible. These scans also enable the visualization of very small lesions in the WM, probably representing nascent WM pathology (green arrows).

**Figure 8:**
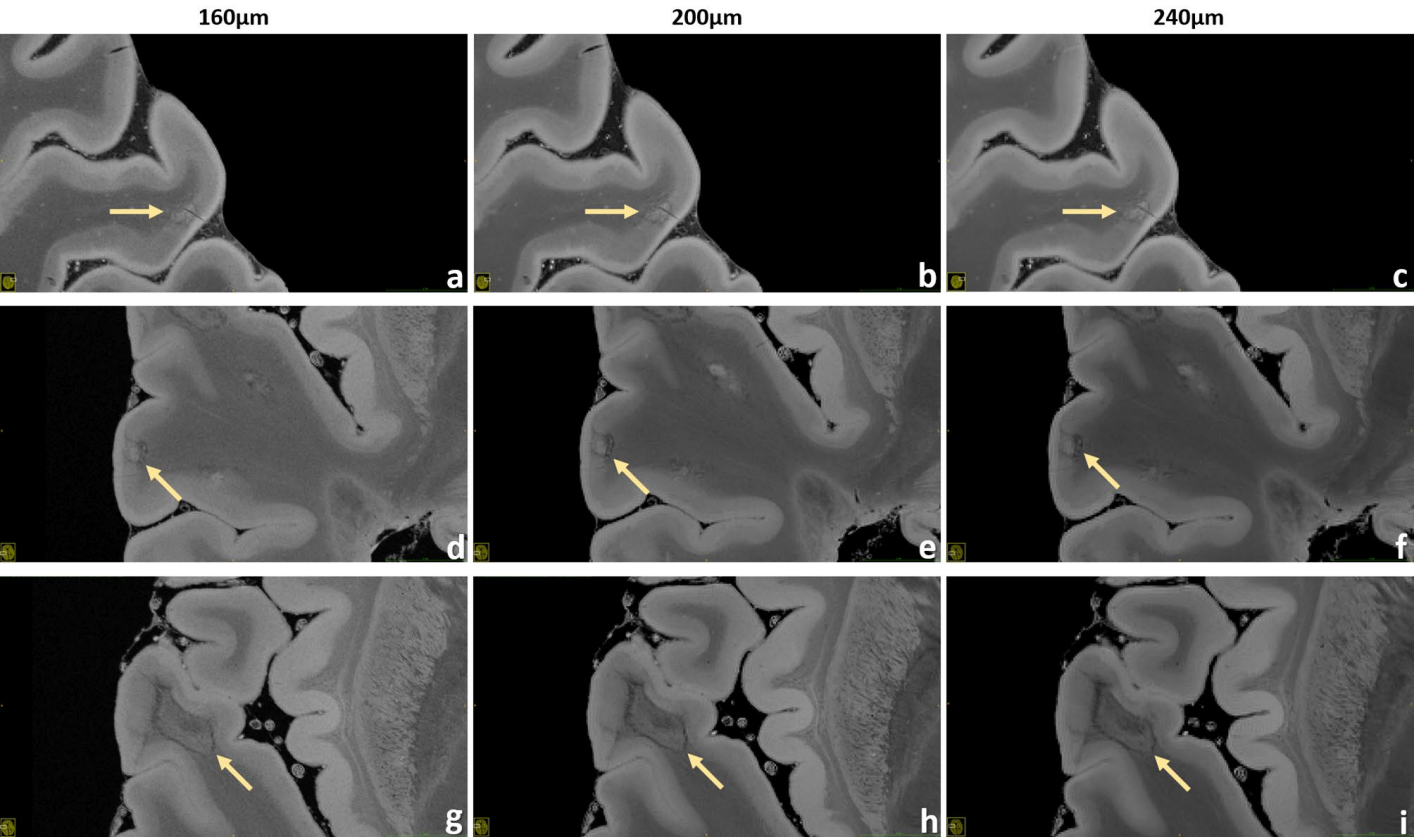
Brain #1, exemplary leukocortical lesions. Upper row: a leukocortical lesion with “central vein” acquired at 160 µm (**a**), 200 µm (**b**) and 240 µm (**c**) resolution is presented. This inhomogeneous area is almost completely contained in the cortex and presents hypointense borders. The “central vein”, a threadlike hypointensity in the middle of the lesion, can be observed easily. Middle row: a leukocortical lesion with hypointense “rim” at 160 µm (**a**), 200 µm (**b**) and 240 µm (**c**) resolution is displayed. This inhomogeneous area located across the border between WM and GM presents a central hyperintensity surrounded by a hypointense frame. Lower row: a leukocortical lesion with hypointense “rim” limited at the WM part at 160 µm (**g**), 200 µm (**h**) and 240 µm (**i**) resolution is demonstrated. In this case the hyperintense WM part of the lesion is surrounded by an hypointense frame; in the nearby cortex a light focal hyperintensity can be noticed.

**Figure 9:**
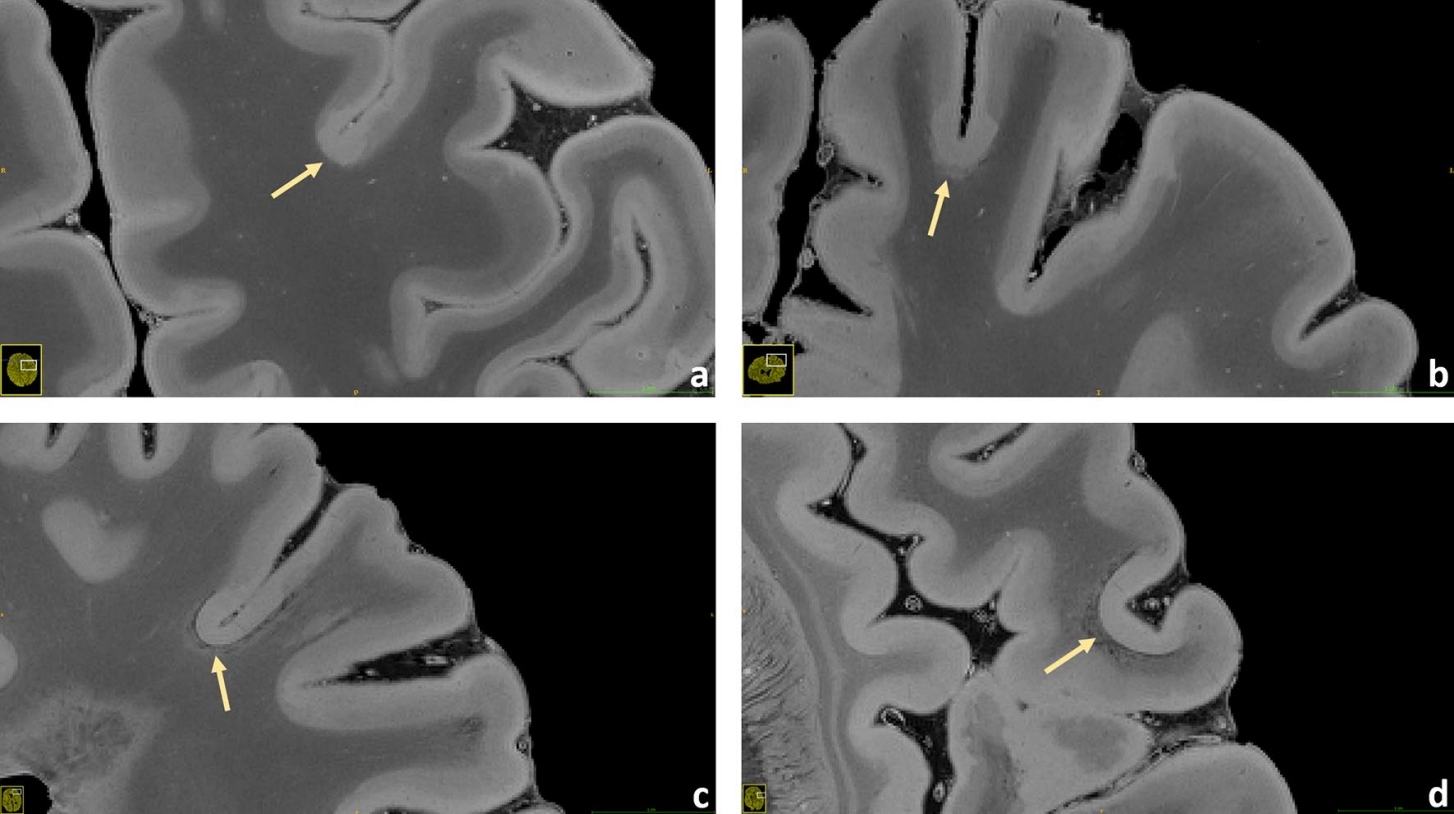
Brain #1. A small potpourri of subpial lesions measured at 200 µm resolution is illustrated (**a**–**d**). In this sequence the cortical structure mostly appears bilayered, probably expressing the different myelin and iron content between the external and the internal layers of the cortex. However, focal losses of this structural organization can be observed well in the pictures: these areas, which do not follow the irroration territory of cortical veins, are especially found in proximity of the sulci; they present as hyperintense throughout the whole cortex with loss of the otherwise well visible bilayered structure. Some of these lesions show a hypointense juxtacortical rim (**c**,**d**).

**Figure 10:**
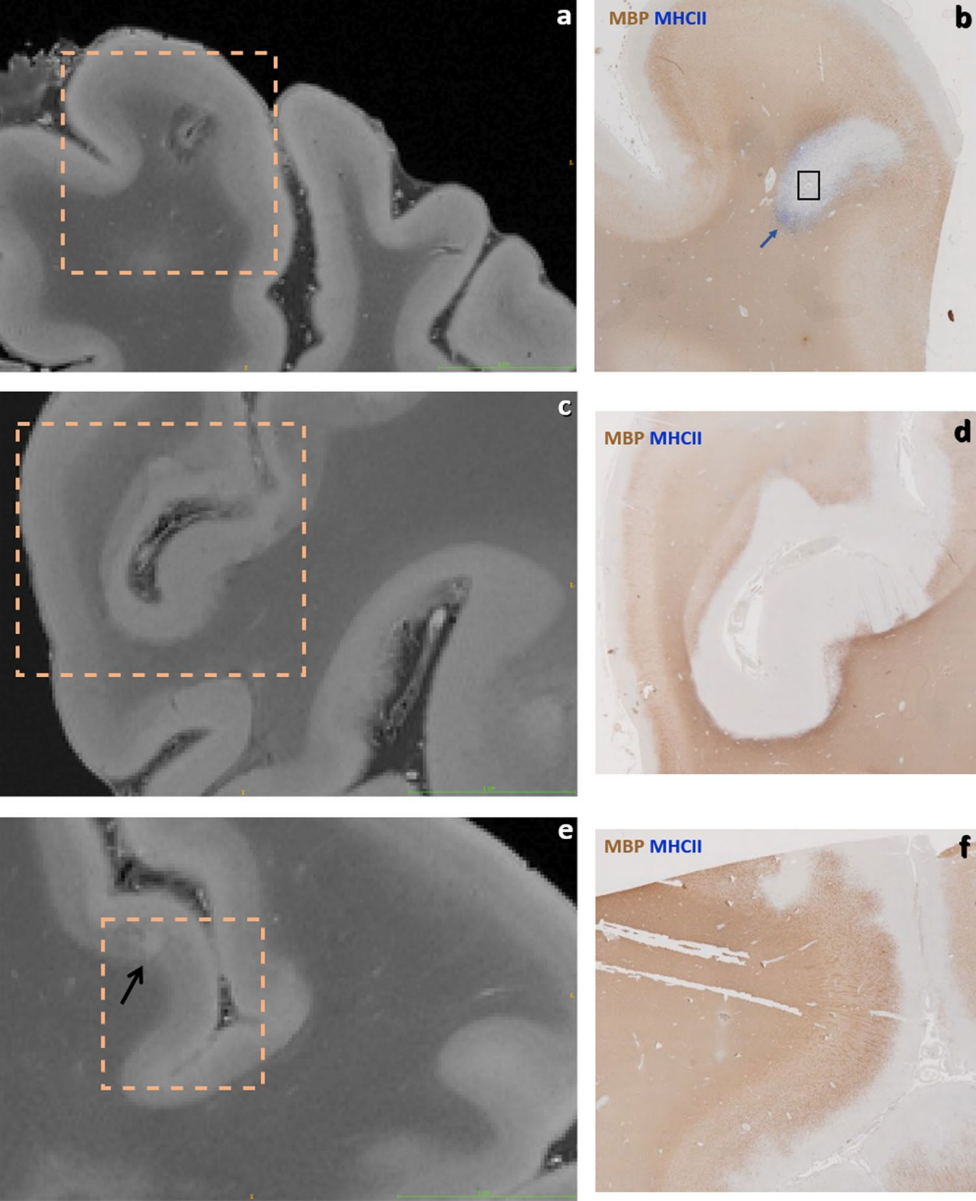
Brain #1, illustration of 200 µm URI-FLASH acquisitions with their associated histology slices (immunohistochemistry for Myelin Basic Protein -MBP- and MHC II). (**a**) Leukocortical lesion with hypointense rim in the white matter and “central vein” sign. (**b**) Corresponding histology slice, showing presence of microglial cells at the lesion border (blue arrow) and a vessel in the middle of the lesion (black rectangle). (**c**) Diffuse and irregular signal alteration (hyperintense) in the cortex at the level of a sulcus. (**d**) The corresponding histology slice shows extended subpial demyelination. (**e**) Small intracortical lesion (black arrow). (**f**) The histological study demonstrates a demyelinated area at the corresponding level.

Generally, a common goal of postmortem MRI is the maximization of the acquired spatial resolution. Using the example of the dentate nucleus, Fig. 2 points out that some structures in the human brain are so hyperfine that even an ultra-high-resolution gain from 240 μm towards 160 µm is still valuable. Figure 3 also shows a comparison for the line of Gennari and for some small lesions. The ultra-high-resolution acquisitions only make sense, however, if the targeted resolution can be realized with “sufficient SNR” within the allotted total acquisition time. Supplementary Table S1 contrasts the minimum total acquisition times (i.e., averages) needed for a desired spatial resolution to obtain such a “sufficient SNR”, as perceived in the eyes of seven clinicians and scientists having long experience with MRI. The 270 µm resolution represents the “fast variant” and it does not necessitate any signal averaging. Supplementary Table S1 also presents the maximum acquisition times per resolution that could be realized within the frame of this work. As a further noteworthy side information, the relative SNRs of the protocols are given.

The individual results of the “minimum SNR survey” are presented in Supplementary Tab. S2. Supplementary Figure S1 provides examples of images at different spatial resolution: acquisitions with the four base protocols of 240 µm, 200 µm, 180 µm, and 160 µm are contrasted with their corresponding averaged acquisitions with both “minimum SNR” and “maximum performed”. Additionally, Supplementary Fig. S2 compares the 200 µm with the lowest 270 µm isotropic resolution.

Figures 4, 5, 6, 7, 8, 9 and 10 display a representative compilation of different small-sized multiple sclerosis lesions: lesions in both the dentate nucleus and the olivary nucleus in the pons, Fig. 4; thalamic and subcortical WM lesions, Fig. 5; pontine and thalamic lesions, Fig. 6; purely intracortical and leukocortical lesions, Figs. 7 and 8; lesions with central vein, Figs. 7 and 8; lesions with hypointense rims, Fig. 8; subpial lesions, Fig. 9; hypointense juxtacortical rim, Fig. 9. These exemplary lesions clearly show that a combination of a good SNR, very-high spatial resolution and strong tissue contrast is essential for delineating these fine structures. Lesions with peculiar hypointense patterns (open and closed rims within the cortex and in the juxtacortical areas) as well as lesions in the gray matter nuclei of the brainstem (i.e. olivary nucleus) could be identified. Furthermore, a very nice correspondence between the signal alterations in the URI-FLASH sequence—especially in gray matter—and the tissue damage at immunohistochemistry was observed (Fig. 10). Additionally, accumulation of microglial cells was detected in areas with overt susceptibility effects (Fig. 10).

Altogether for Figs. 2, 3, 4, 5, 6, 7 and 8, it should be noted how very well equivalent slices of similar geometry could be found in the datasets of different ultra-high resolutions, even though the brain container was completely removed from the MR system in between: the acquisitions were independent with at least 1 week apart.

Supplementary Figures S3 to S6 present further examples for the morphology of both brain #1 and #2 in different reformations.

Finally, the Supplements contain three videos that browse through the entire MS brain #1 with a 200 µm resolution in different slice directions.

## Discussion

### Technical and methodological aspects

We developed an ex vivo whole-brain MR imaging approach that facilitates isotropic 3D ultra-high-resolution imaging up to 160 μm with a strong soft tissue contrast on a common clinical 3 T MR system. By utilizing only standard hardware components and online image reconstruction (i.e., directly on the MR system), the rationale was to test the viable boundaries for ex vivo URI at 3 T and, consequently, establishing an MRI concept that can be well reproduced on other MR systems. Thus, our approach was substantially different from that of a recently published work^4^, where a spatial resolution of 100 μm was achieved in a brain specimen; the latter employed a similar acquisition sequence but made use of 7 T field strength, of a custom-made phased-array coil as well as of self-made computational tools that allow bypassing the raw data storage capacity of the MR system, did offline MRI reconstruction and enable post-processing of a few terabytes of MR raw data^4^.

Five base protocols of 160 µm, 180 µm, 200 µm, 240 µm, and 270 µm isotropic resolution were established. Acquisition times between approximately 7 h up to 90 h were investigated. Even the “low” 270 μm base protocol offers great potential for 7 h acquisition time; this protocol can be easily added to other postmortem examinations. As our data show, the full capabilities of a 160 μm acquisition are revealed for our ex vivo approach covering at least ca. 42 h—the recommended minimum acquisition time based on Supplementary Tab. S1, which can be usually organized well in the light of routine measurements at site by utilizing weekends.

In the following, different physical as well as methodological aspects of the presented ex vivo URI-FLASH approach are discussed and important technical considerations as well as trade-off decisions are depicted. Apart from its technical feasibility key factors for the development have been acquisition time and SNR. For an MRI sequence in 3D Cartesian acquisition mode the total acquisition time can be directly determined as

1 $$TA=avg\cdot {N}_{PE}\cdot {N}_{3D}\cdot TR,$$

where *avg* is the number of signal averages performed, *TR* the sequence’s repetition time and *N*_PE_ and *N*_3D_ are the numbers of measured phase-encoding steps of the two corresponding phase encoding directions. In contrast, the SNR is a quite complex physical quantity with many contributing factors that are partially interdependent. For the present purpose the average SNR of a voxel may be approximated as

2 $${\langle SNR\rangle }_{voxel} \sim {B}_{0}\cdot\Delta V\cdot Coil\left(\cdots \right)\cdot Seq\left(\cdots \right)\cdot \frac{\sqrt{avg}}{\sqrt{BW} },$$

where *B*_0_ is the main magnetic field strength, Δ*V* is the voxel volume, $Coil\left(\cdots \right)$ represents a net signal effect related to RF receiver coil properties, $Seq\left(\cdots \right)$ depicts the MRI sequence’s signal generation, and *BW* denotes the receiver bandwidth. For a constant voxel volume Δ*V* it is also

3 $$\Delta V=const: \:\: {\langle SNR\rangle }_{voxel} \sim \sqrt{{N}_{PE}\cdot {N}_{3D}}\: .$$

Again, *N*_PE_ and *N*_3D_ are the numbers of measured(!) phase-encoding steps here.

On purpose, Eq. (2) and the term $Seq\left(\cdots \right)$ in specific do not consider signal effects of MR tissue properties like the relaxation time T2 that generates MRI contrast—this is presumed as desired and necessary. Similarly, all effects of different *k*-space sampling schemes are neglected: without applying any corrections, “Cartesian” is the most robust *k*-space sampling in terms of long-term stability. Furthermore, any non-Cartesian scheme would probably require offline reconstruction and, thus, raw data exports. Since the raw data export of only one 160 μm URI-FLASH base protocol measurement already requires 189 GB of disc space, overall, “simple Cartesian” was the *k*-space sampling scheme of choice.

Generally, Eq. (2) emphasizes the well-known fact that increasing MRI resolution in 3D can lead to dramatic losses in SNR; and if these losses are meant to be compensated by signal averaging, a quadratic and not linear increase of TA results, which renders “averaging” inefficient for higher numbers of averages. With *B*_0_ = 3 T, $Coil\left(\cdots \right)$ being the standard head and neck coil (cf. below) and signal averaging being more the “last solution”, the focus for potential SNR gain compared to the in vivo situation lies on the acquisition sequence $Seq\left(\cdots \right)$ including its receiver bandwidth *BW*.

In principle, the ex vivo URI-FLASH approach is based on five major reasonings that are related to either the term $Seq\left(\cdots \right)$ or to the bandwidth *BW* or both. In brief: (1) we experienced that RF spoiled gradient-echo (“FLASH”) sequences, which are also harnessed for quantitative susceptibility mapping^19^, facilitate acquiring high-quality MR images with remarkable soft-tissue contrast under ex vivo conditions; (2) the T1 relaxation times are considerably shortened in fixed ex vivo tissue^20^, which leads to a much faster recovery of longitudinal magnetization. RF spoiled gradient-echo sequences with short repetition time TR, demonstrating steady state magnetization response^21,22^, benefit a lot from this effect: they can be tuned to a much higher signal efficiency compared to in vivo conditions; (3) an exceptionally low receiver bandwidth of 50 Hz/Pixel was used to enhance the overall SNR considerably and to optimize the time the MR system spends on signal acquisition for the given echo time TE and repetition time TR. Additionally, it enabled comparatively low gradient strengths and moments for high spatial resolutions. (4) An in-house FLASH sequence was utilized that is less restricted in terms of MR parameter ranges and internal software architecture (cf. Methods section). (5) The full value for basic and clinical neuroscience is obtained for isotropic 3D resolutions alone: only then an unbiased view is possible, effects like partial volume are invariant of the direction, and multi-planar reformations are feasible in arbitrary directions without loss of information.

Further optimizations for the FLASH sequence were to maximize the tissue SNR by choosing the excitation flip angle close to the expected Ernst angle^23^; that is why the flip angle changes minorly with the different protocols: it depends on TR (cf. Methods section). Another possibility would have been to acquire multiple gradient echoes for averaging them up to increase SNR. But on the contrary, only one echo with an exceedingly low bandwidth of 50 Hz/pixel was acquired, which is equivalent to acquire signal for a very long duration. Compared to the multiple echo solution, the low bandwidth solution has the benefits that less gradient switching is necessary, and that lower gradient strengths and lower gradient moments are required in the readout direction, which are also technically limited. So, for the ex vivo situation, where very high spatial resolutions are targeted, the bandwidth variant is of advantage. Two potential drawbacks of the low bandwidth solution are a large chemical shift effect and an increased sensitivity to background gradients; however, because of the lack of fatty tissue and of the fact that the brain is immersed in almost susceptibility-matched perfluoropolyether—in addition to applying a good shim—those drawbacks did not cause any issue.

Neglecting any contrast, since steady-state based sequences are so advantageous regarding their acquisition efficiency in ex vivo conditions, a self-evident question may be why not employing a fully balanced steady state free precession (b-SSFP or TrueFISP) sequence. This would warrant an even higher signal level and improved acquisition efficiency, increasing the term $Seq\left(\cdots \right)$, which could be directly translated into quite shorter acquisition times due to less averaging (Eqs. 1, 2). Generally, b-SSFP sequences are prone to susceptibility effects and their so-called banding artifacts. Furthermore, simply put, they require high bandwidths, fast gradient switching and high RF pulse power for a good performance. Additionally, they are more sensitive to long-term drift effects compared to the RF spoiled variant FLASH. All these properties are counterproductive for realizing URI on clinical MR systems (cf. technical issues below).

That the choice of the acquisition sequence and its overall efficiency and sampling is of notable importance for URI conditions, can also be seen by comparing the URI-FLASH based “fast protocol” achieving an isotropic 270 µm spatial resolution in 7 h to the SPACE protocol applied to a whole postmortem brain at 3 T, obtaining “only” an isotropic 400 µm resolution in very similar TA = 7.5 h^5^. Acquiring a (400/270)^3^ = 3.3 times smaller voxel volume compared to^5^ generally results in a 3.3 times lower SNR (Eq. 2). So, compensating this SNR loss solely by signal averaging would require a 3.3^2^ ≅ 11 times longer acquisition time then (Eq. 2).

Although this rough calculation is just volume-based and neglects spatial encoding and also differences in head-coils^5^, it already shows the higher URI-FLASH acquisition efficiency compared to the SPACE one. In fact, for the SPACE^24^ sequence, being a specialized 3D variant of the turbo spin echo sequence with low refocusing flip angles^25^, the considerably shortened T1 and T2 relaxation times under ex vivo conditions are a clear disadvantage for both acquisition efficiency and image quality in terms of image blurring^5,26^. Moreover, receiver bandwidths as low as 50 Hz/Pixel would have a further detrimental effect on the SPACE sequence’s image quality.

As discussed above, increasing the isotropic 3D spatial resolution in MRI comes at the expense of considerable SNR reduction and retrieving SNR via signal averaging is time-consuming (cf. Eq. 2). Therefore, on one hand, it may be worthwhile to limit the number of averages to the absolute minimum. On the other hand, even a moderate noise impact may hamper the resolving of very fine structures, as it can be found in the basal ganglia or in the cerebellum. As a matter of fact, the current 270 µm URI-FLASH protocol is roughly the break-even point in terms of SNR: protocols of even higher resolutions necessitate signal averaging (cf. Supplementary Tab. S2). Exactly for this reason, the URI-FLASH protocols do not utilize any common acceleration technique like GRAPPA, SENSE, Partial Fourier, or compressed sensing: any reduction of measured phase encoding steps leads to both shorter acquisition time (Eq. 1) but also to unwanted SNR reduction if the voxel volume, i.e., the 3D resolution is meant to be constant (Eq. 3).

To investigate the image quality after averaging for the four highest spatial resolutions, a small survey was organized: seven clinicians and scientists with experience in brain MRI evaluated respective series of images with increasing averages, judging about the displayed SNR and overall image quality. As a result, resolution-specific recommendations for a minimum of averages (i.e., minimum total acquisition time) perceived to be essential for “sufficient SNR” were defined for the four highest resolutions that necessitate signal averaging (Supplementary Tab. S1, individual decisions in Supplementary Tab. S2). A visual comparison of the resulting decisions is presented in Supplementary Fig. S1. With the aid of Supplementary Tab. S1 and Supplementary Fig. S1 the reader can orientate on which ultra-high-resolution protocol to choose for a given, available total acquisition time. Moreover, Figs. 2, 3, 4, 5, 7 and 8 as well as Supplementary Fig. S1 combine images of different spatial resolutions such that the reader can get a better notion of possible differences and what image quality can be expected for a given acquisition time, but also presenting a variety of locations and lesions at the same time.

The targeted resolutions require acquisition times of hours to days on a clinical MR system, where the whole gradient and RF hardware components may be permanently operated at maximum performance: this situation represents an extreme strain on the hardware components, which can even lead to their damage or failure. Moreover, factors like maximum gradient strength, maximum gradient power amplifier duty cycle, and maximum RF power amplifier duty cycle put restrictions to the maximal resolution and acquisition efficiency. A substantial benefit of the used URI-FLASH approach is that its strain on the hardware is low despite the requirements: the exceptionally low bandwidth leads to relatively weak gradients and gradient moments on the readout axis. Furthermore, with $\mathrm{TR}/2 \cong \mathrm{TE}=18\mathrm{ms}$, enough time is left for the phase and 3D encoding gradients in the sequence. Altogether, this facilitates the ultra-high resolutions we could achieve and holds mechanical vibrations in the MR system at bay. Naturally, strong mechanical vibrations affect URI adversely and can even stimulate increased helium boil-off if the mechanical vibrations hit certain eigen frequencies of the MR system. Similarly, the strain on the RF hardware is also in the normal range for URI-FLASH, since only one RF excitation pulse of low flip angle every $\mathrm{TR}\approx 37\mathrm{ms}$ is applied.

Manufacturer-supplied MR sequences limit which MR parameters can be influenced and particularly their range of values. Ex vivo MRI often requires parameter combinations and values, however, which are uncommon in clinical in vivo MRI. Consequently, we programmed a basic RF spoiled gradient echo (FLASH) sequence that allows broad parameter ranges and bypasses part of the internal software like view-ordering schemes. As a result, we could avoid non-desirable limitations for parameters such as base matrix size and maximal product of phase encoding steps in 3D. Additionally, it was made sure that all gradient durations were stretched to the full possible limit, thereby reducing the gradient amplitude (‘natural’ exclusions: readout and slab selection gradient). Thus, neglecting any acquisition time for the moment, eventually, our URI-FLASH approach was strictly limited by the maximal image reconstruction size supported by the MR system: at 160 µm isotropic resolution together with the 20-channel head coil, it was just feasible to spatially encode the full human ex vivo brain. At last, to avoid any conflict with the limited raw data storage space on the MR system, single acquisitions were performed first and averaged up manually at a later timepoint (cf. Supplementary Fig. S1).

In line with our general rationale of establishing a reproducible ex vivo URI approach on a common 3 T MR system, we employed the standard 20-channel phased-array head and neck coil supplied by the manufacturer. On the one hand, the spaciousness of this coil helps to accommodate the ad-hoc brain container. Moreover, it provides a quite homogeneous B_1_ reception field, which—in combination with the quite homogeneous B_1_^+^ excitation field of the 3 T MR system—leads to MR images of overall homogeneous tissue intensity and contrast. A comparison of the presented images with images of Ref.^4^ substantiate this observation. A further advantage is that fewer coil elements necessitate less reconstruction memory on the MR system; thus, since we utilized all the available MR scanner host resources, the maximal resolution that could be reconstructed depended on the number of coil elements. On the other hand, a drawback of the 20-channel head and neck coil is the notably lower SNR achieved compared to an alternative vendor coil with more coil elements or a custom-made coil.

### Clinical aspects

Figures 1, 2, 3, 4, 5, 6, 7, 8, 9, 10 and Supplementary Figs. S1 to S6 all underline the potential of 3 T based ex vivo examinations in clinical neuroscience.

First, URI-FLASH allows to distinguish different layers of the cerebral cortex, namely, the external part of the cortex appears more hyperintense, most probably representing the subpial molecular layer and the external part of the granular layer (Figs. 3, 5, 7, 8, 9). Furthermore, the line of Gennari, in the calcarine fissure, is hypointense (Fig. 3). Second, because of its tissue-sensitive contrast and the sensitivity to susceptibility effects, URI-FLASH also permits the identification of some intrathalamic nuclei like the pulvinar nuclei and the medial geniculate nucleus (Fig. 1b,c) as well as the medio dorsal nucleus, anterior nucleus and the *lamina intermedia* (Supplementary Fig. S3).

Based on the comparison of URI images with their corresponding histological slices, we also showed how URI-FLASH enables a fine delineation of the MS demyelinating pathology, especially in the cortex. In fact, URI-FLASH appears to be very sensitive to both the identification of subpial lesions which are the most specific and frequent cortical lesion subtype in MS patients^27,28^ and to the identification of very small intracortical lesions, especially at the highest resolutions such as 160 µm and 200 µm.

Identifying cortical demyelinated lesions with MRI is challenging due to the small change in myelin-related signal that is observed in those lesions compared to WM lesions. Besides, cortical lesions are often less inflammatory than WM lesions^29^, leading to limited alteration of signal intensity in T1 and T2 weighted MRI^30,31^. URI-FLASH at 3 T shows therefore not only the advantage of a very high micrometric spatial resolution but also to the subtle demyelinating process occurring in cortical lesions in MS patients, as previously shown at 7 T^32^.

Besides, the contrast provided by URI-FLASH—which combines an exquisite sensitivity to the presence of paramagnetic substances such as iron—allows to identify multiple characteristics of both cortical and white matter MS lesions, such as the presence of complete iron rims surrounding leukocortical lesions (Fig. 8d–f) or just their white matter part (Fig. 8g–i). Lesions harboring paramagnetic rims, which reflect the accumulation of microglia cells and ongoing smoldering inflammatory activity^33,34^, are particularly important as they have been linked to disease progression and high disability in MS patients^33,35^. Hence, URI-FLASH may provide a new window to understand the role of those lesions in the cortical layers and in the juxtacortical white matter.

Likewise, URI-FLASH provides evidence of gradients of tissue pathology, both in WM and in GM, which are clearly paralleling the patterns described in neuropathological studies^27,36,37^.

The micrometric resolution of URI-FLASH images also provides a clear advantage for the study of nascent sub-millimetric pathology in WM (Fig. 7), small punctiform lesions in cortical GM (Fig. 7) and lesions affecting the cerebellar nuclei and the convoluted GM/WM cortical layers of the cerebellar cortex (Fig. 4) and the deep GM nuclei (Fig. 1, Supplementary Fig. S3).

### Relevance of the work

The presented URI-FLASH approach provides a microscopic insight of the entire human brain at 3 T, which is achieved through a micrometric resolution, a tissue-specific contrast and a superb sensitivity to susceptibility effects. URI-FLASH is therefore an excellent method to investigate the microscopic characteristics of the human brain as well as its changes in a complex pathology like multiple sclerosis, especially in regions that require an ultra-high spatial resolution such as the cortex and the small-sized gray matter nuclei of the thalamus and the brainstem. This work opens the perspective to (1) investigate the global impact of MS pathology and of its variants; (2) perform postmortem imaging in largely available scanner types and with standard hardware; and (3) provide new educational tools for neuroanatomy and neuropathology, in case brain specimens are not available.

## Supplementary Information

Supplementary Information 1.

Supplementary Information 2.

Supplementary Information 3.

Supplementary Video 1.

Supplementary Video 2.

Supplementary Video 3.

## Data Availability

The datasets generated during and/or analyzed during the current study are available from the corresponding author on reasonable request.

## Abbreviations

URI: Ultra-high-resolution imaging
FLASH: Fast low-angle shot
GM: Gray matter
WM: White matter
