# Supplementary material for: Imaging multiple sclerosis pathology at 160 μm isotropic resolution by human whole-brain ex vivo magnetic resonance imaging at 3 T
Source: Sci Rep. 2021 Jul 29;11:15491. doi: 10.1038/s41598-021-94891-1 (PMC8322069; doi:10.1038/s41598-021-94891-1)
Supplement: Supplementary file 1 — Supplementary Information 1. [file 41598_2021_94891_MOESM1_ESM.pdf]

SUPPLEMENTARY INFORMATION: TABLES

Imaging Multiple Sclerosis Pathology at 160µm Isotropic Resolution by Human Whole-Brain *Ex Vivo* Magnetic Resonance Imaging at 3T

Weigel, Matthias, PhD [1,2,3,4,*]; Dechent, Peter, PhD [5]; Galbusera, Riccardo, MD [1,2,3]; Bahn, Erik, MD [6]; Nair, Govind, PhD [7]; Lu, Po-Jui, MS [1,2,3]; Kappos, Ludwig, MD [1,2,3]; Brück, Wolfgang, MD [6]; Stadelmann, Christine, MD [6]; Granziera, Cristina, MD, PhD [1,2,3]

[1] Translational Imaging in Neurology (ThINk) Basel, Department of Biomedical Engineering, University Hospital Basel and University of Basel, Gewerbestrasse 14, 4123 Allschwil, Switzerland

[2] Neurologic Clinic and Policlinic, Departments of Medicine, Clinical Research and Biomedical Engineering, University Hospital Basel and University of Basel, Petersgraben 4, 4031 Basel, Switzerland

[3] Research Center for Clinical Neuroimmunology and Neuroscience Basel, University Hospital Basel and University of Basel, Petersgraben 4, 4031 Basel, Switzerland

[4] Division of Radiological Physics, Department of Radiology, University Hospital Basel, Petersgraben 4, 4031 Basel, Switzerland

[5] Department of Cognitive Neurology, MR-Research in Neurosciences, University Medical Center Göttingen, Robert-Koch-Strasse 40, 37075 Göttingen, Germany

[6] Institute of Neuropathology, University Medical Center Göttingen, Robert-Koch-Strasse 40, 37075 Göttingen, Germany

[7] Quantitative MRI core facility, National Institute of Neurological Disorders and Stroke, National Institutes of Health, 10 center drive, Bethesda, Maryland, USA

***Correspondence: Matthias Weigel (matthias.weigel@unibas.ch)**

Supplementary Table S1: Overview of Acquisition Times for the Investigated Protocols and Brains

**Isotropic 3D resolution:**	**270 µm**	**240µm**	**200µm**	**180µm**	**160µm**
**$SNR_{relative}$ / a.u.**	1.00	0.78	0.42	0.28	0.19
**TA_{base} / h**	07:00:31	09:08:07	08:12:33	07:04:49	06:57:20
**$avg_{min-SNR}$ #**	*N.N.*	3	4	5	6
**$TA_{min-SNR}$ / h**	07:00:31	27:24:21	32:50:12	35:24:05	41:44:00
**MS brain #1 $avg_{max-performed}$ #**	-	9	11	8	11
**$TA_{max-performed}$ / h**	-	82:13:03	90:18:03	56:38:32	76:30:40
**MS brain #2 $avg_{max-performed}$ #**	*N.N.*	-	8	-	-
**$TA_{max-performed}$ / h**	07:00:31	-	65:37:44	-	-

$SNR_{relative}$: Relative SNR of the different acquisition protocols.

TA_{base}: Acquisition time of the base protocol (single acquisition without averaging).

$avg_{min-SNR}$: Minimum number of averages recommended for "sufficient SNR", see Supp. Tab. S2 as well as Materials and Methods section.

$TA_{min-SNR}$: Resulting acquisition time for the recommended "minimum SNR protocol".

$avg_{max-performed}$: Maximum number of averages that could be performed within the frame of the investigations.

$TA_{max-performed}$: Resulting acquisition time for maximum number of averages performed.

"*N.N.*": not necessary.

Manual averaging of the acquired magnitude images from repeated base protocol measurements.

Supplementary Table S2: Overview of Expert Decisions for the Visual SNR Evaluation on MS Brain #1

**Recommendations:**	**240µm**	**200µm**	**180µm**	**160µm**	**Expertise**
**averages$_{min-SNR}$ (E1) [#]**	1	3	3	5	Neurologist
**averages$_{min-SNR}$ (E2) [#]**	6	4	5	6	Neuroradiologist
**averages$_{min-SNR}$ (E3) [#]**	1	2	2	2	Neurologist
**averages$_{min-SNR}$ (E4) [#]**	4	5	7	6	Image Processing Expert
**averages$_{min-SNR}$ (E5) [#]**	3	6	6	9	MRI specialist (Biologist)
**averages$_{min-SNR}$ (E6) [#]**	1	1	3	4	Neurologist
**averages$_{min-SNR}$ (E7) [#]**	3	5	6	8	Physicist
**arithmetic MEAN**	**2.7**	**3.7**	**4.6**	**5.7**	---
**MEDIAN**	**3**	**4**	**5**	**6**	---

averages$_{min-SNR}$: Minimum number of averages recommended for “sufficient SNR”, here, listed by expert-rater who intentionally have different expertise, i.e., different clinical and scientific backgrounds.

[#] Manual averaging of the acquired magnitude images from repeated base protocol measurements.
