# Supplementary material for: Imaging multiple sclerosis pathology at 160 μm isotropic resolution by human whole-brain ex vivo magnetic resonance imaging at 3 T
Source: Sci Rep. 2021 Jul 29;11:15491. doi: 10.1038/s41598-021-94891-1 (PMC8322069; doi:10.1038/s41598-021-94891-1)

SUPPLEMENTARY INFORMATION: FIGURES

Imaging Multiple Sclerosis Pathology at 160µm Isotropic Resolution by Human Whole-Brain *Ex Vivo* Magnetic Resonance Imaging at 3T

Weigel, Matthias, PhD [1,2,3,4,*]; Dechent, Peter, PhD [5]; Galbusera, Riccardo, MD [1,2,3]; Bahn, Erik, MD [6]; Nair, Govind, PhD [7];

Lu, Po-Jui, MS [1,2,3]; Kappos, Ludwig, MD [1,2,3]; Brück, Wolfgang, MD [6]; Stadelmann, Christine, MD [6]; Granziera, Cristina, MD, PhD [1,2,3]

[1] Translational Imaging in Neurology (ThINk) Basel, Department of Biomedical Engineering, University Hospital Basel and University of Basel, Gewerbestrasse 14, 4123 Allschwil, Switzerland

[2] Neurologic Clinic and Policlinic, Departments of Medicine, Clinical Research and Biomedical Engineering, University Hospital Basel and University of Basel, Petersgraben 4, 4031 Basel, Switzerland

[3] Research Center for Clinical Neuroimmunology and Neuroscience Basel, University Hospital Basel and University of Basel, Petersgraben 4, 4031 Basel, Switzerland

[4] Division of Radiological Physics, Department of Radiology, University Hospital Basel, Petersgraben 4, 4031 Basel, Switzerland

[5] Department of Cognitive Neurology, MR-Research in Neurosciences, University Medical Center Göttingen, Robert-Koch-Strasse 40, 37075 Göttingen, Germany

[6] Institute of Neuropathology, University Medical Center Göttingen, Robert-Koch-Strasse 40, 37075 Göttingen, Germany

[7] Quantitative MRI core facility, National Institute of Neurological Disorders and Stroke, National Institutes of Health, 10 center drive, Bethesda, Maryland, USA

***Correspondence: Matthias Weigel (matthias.weigel@unibas.ch)**

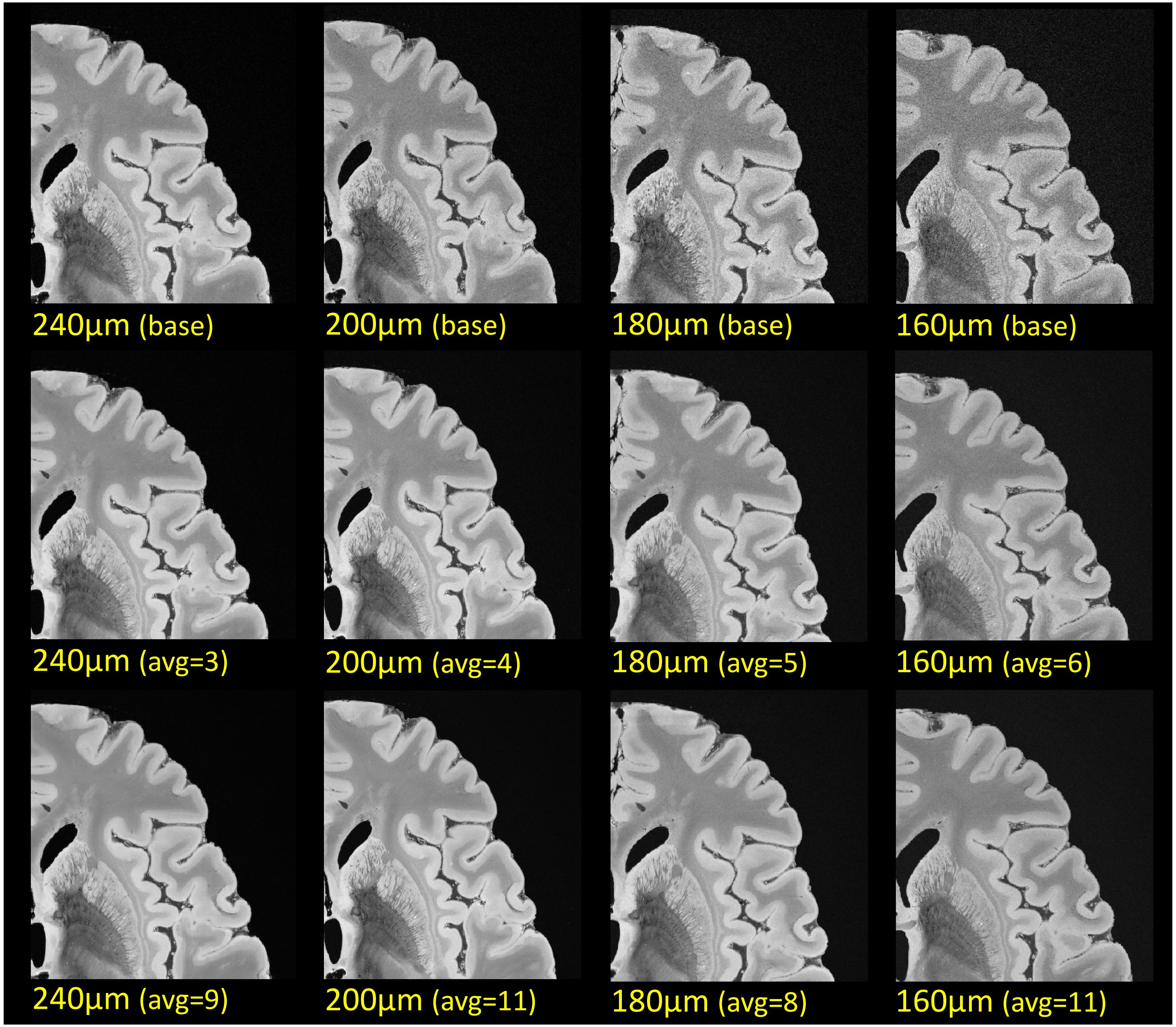

Suppl. Figure S1:

Contrasting juxtaposition of the four highest resolutions and the effect of averaging (brain #1). Generally, only quarter images are presented to improve the recognition of SNR changes; the window-levelling is intentionally ‘bright’.

Upper row: Display of the single acquisitions as specified (base protocol, i.e., no averaging).

Middle row: Averaged acquisitions with a “minimum number of averages” to obtain “sufficient SNR for investigation and diagnosis” at the respective resolution – based on a survey under experienced clinicians and scientists, see Suppl. Tabs. S1 and S2.

Lower row: Corresponding images with the maximal number of averages that could be performed within the frame of this work.

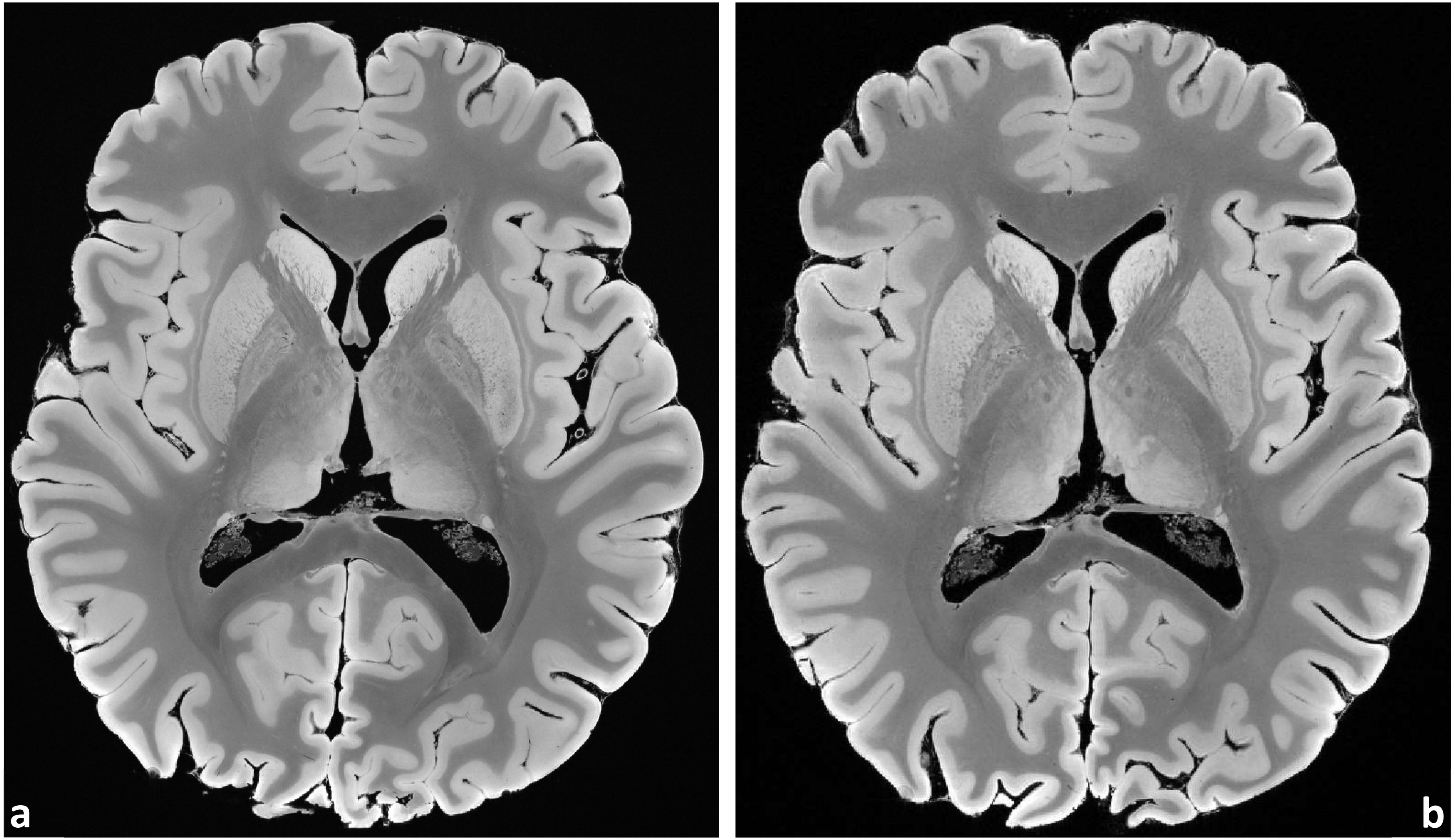

Suppl. Figure S2:

Brain #2, comparison of the 200µm acquisition (**a**) with the “fast variant” URI-FLASH 270µm (**b**), which does not require any signal averaging. Depicted are similar slices of similar orientation in each native space.

(Like for all other mutual MRI comparisons, here, no co-registration methods were used.)

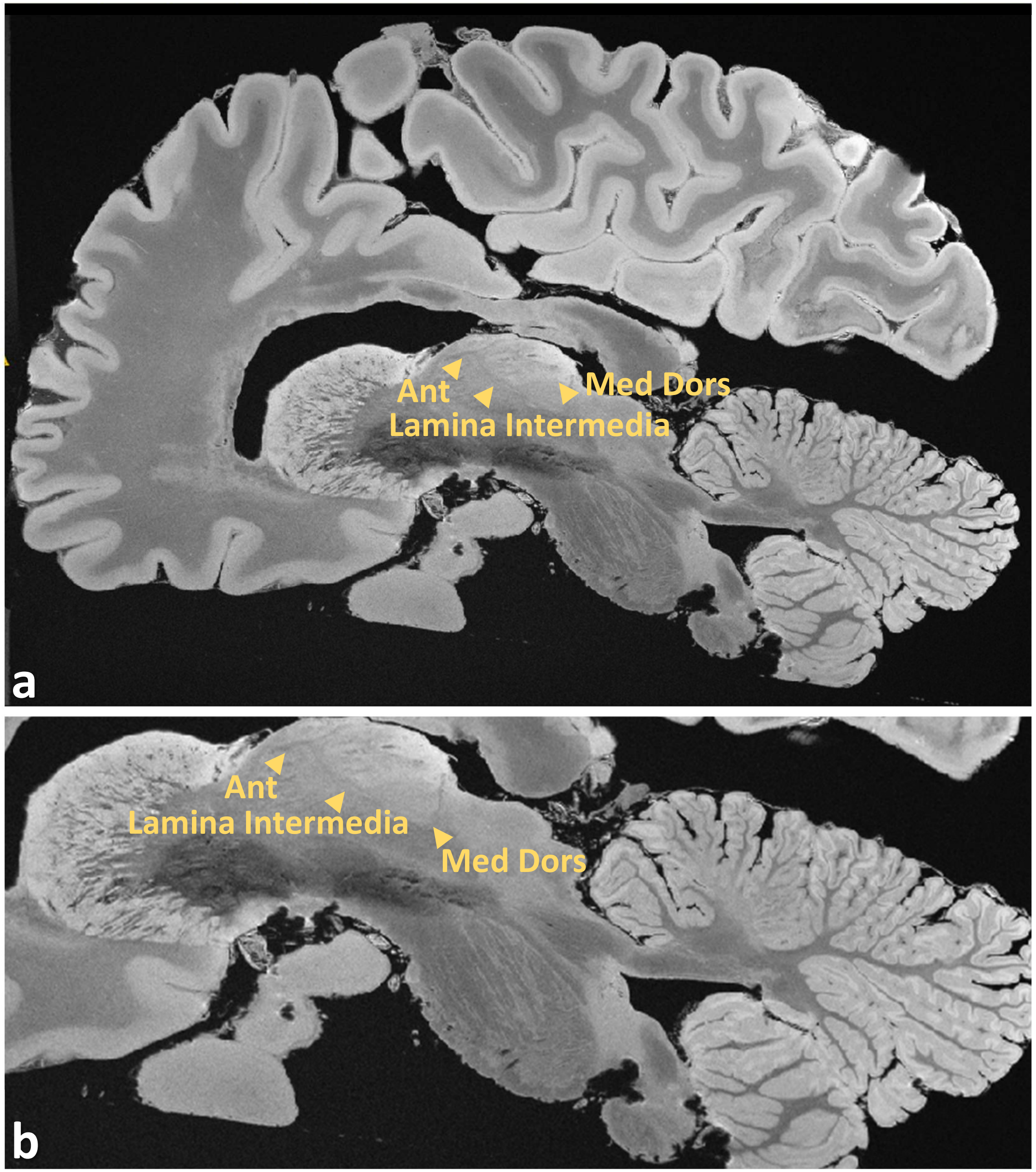

Suppl. Figure S3:

Not least due to the full isotropic 3D resolutions, the sagittal reformations of the MRI datasets also reveal fascinating details (brain #1): A 160µm cross-section (**a**) with an enlargement of the basal ganglia, the thalamus with its nuclei, the brainstem and the cerebellum (**b**) are shown. Gold arrowheads mark the thalamic nuclei as follows: medio dorsal (*Med Dors*), anterior (*Ant*), *lamina intermedia*, and medial geniculate nucleus.

The two subfigures also display in unprecedented detail the cerebellum's *arbor vitae* at 3T field strength.

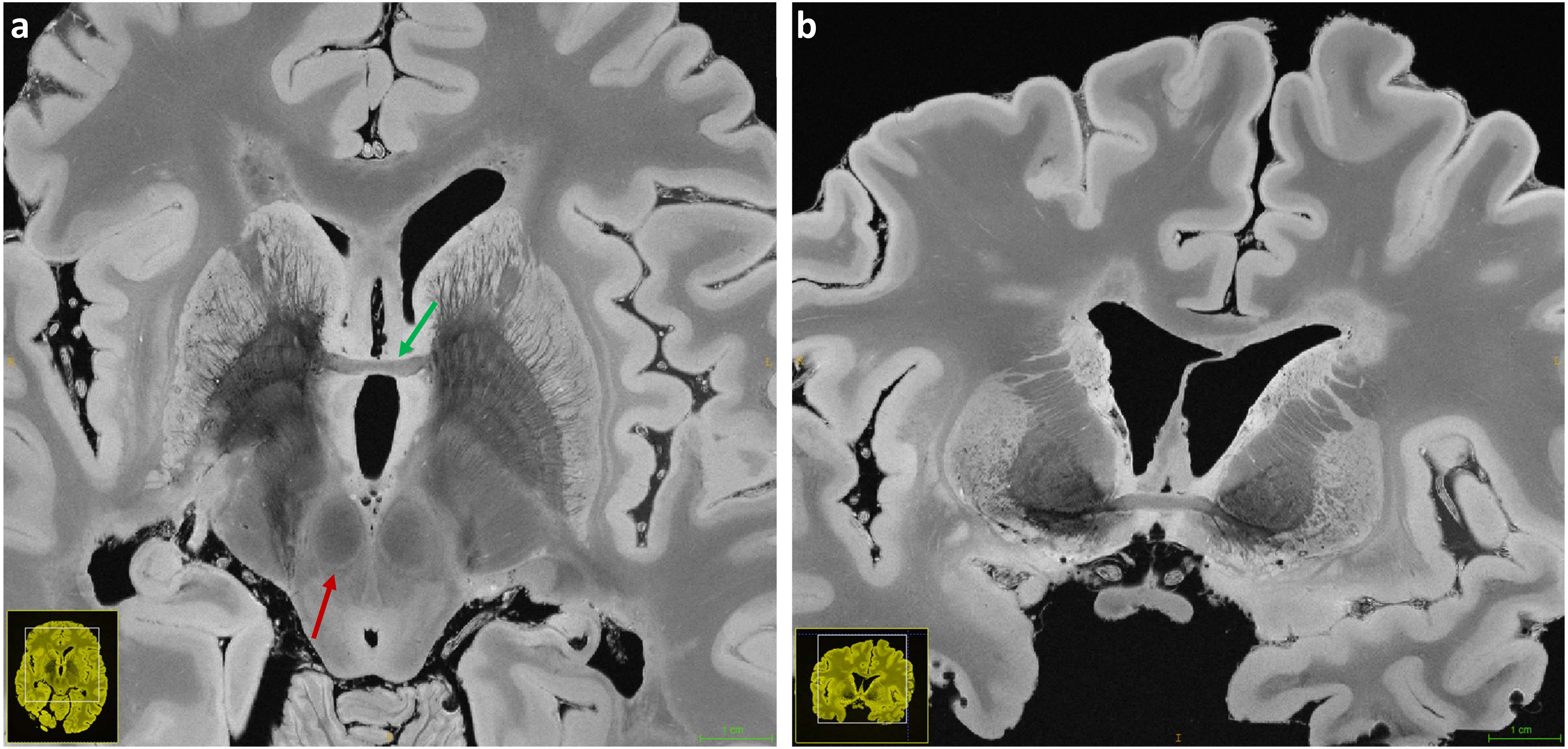

Suppl. Figure S4: Brain #1, zoomed views of a transverse section at 180µm resolution (**a**) and of a coronal reformation of the 240µm dataset in the plane of the anterior commissure are presented (**b**). Structures like the basal ganglia and its venous network as well as the claustrum and the anterior commissure (green arrow) are easily recognized. In the transverse plane the red nuclei are very well defined (red arrow).

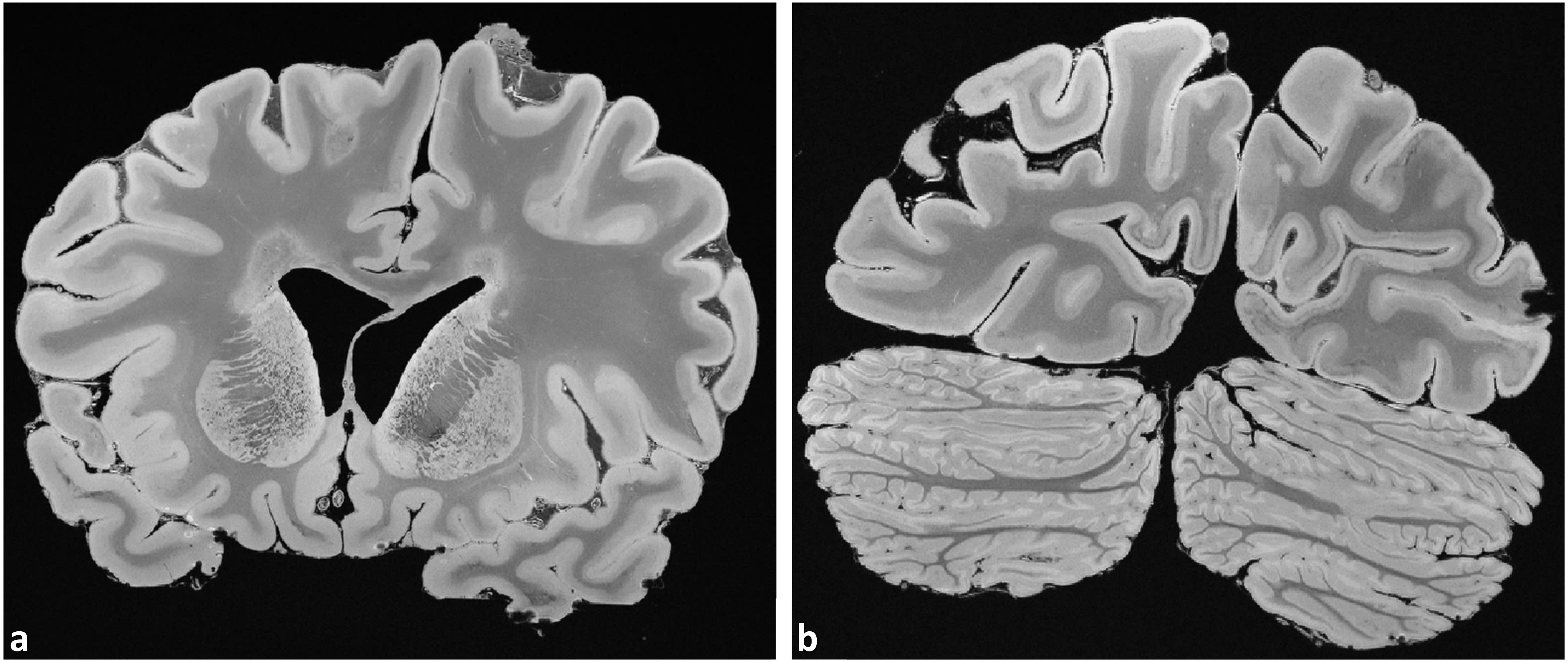

Suppl. Figure S5:

Representative coronal sections of brain #1 from the 200µm (**a**) and the 160µm (**b**) resolution dataset are shown. The interconnected structure of the striatum nuclei (i.e., putamen and caudate) (**a**) and the very tightly folded layers of GM of the cerebellar cortex (**b**) can be visualized in detail.

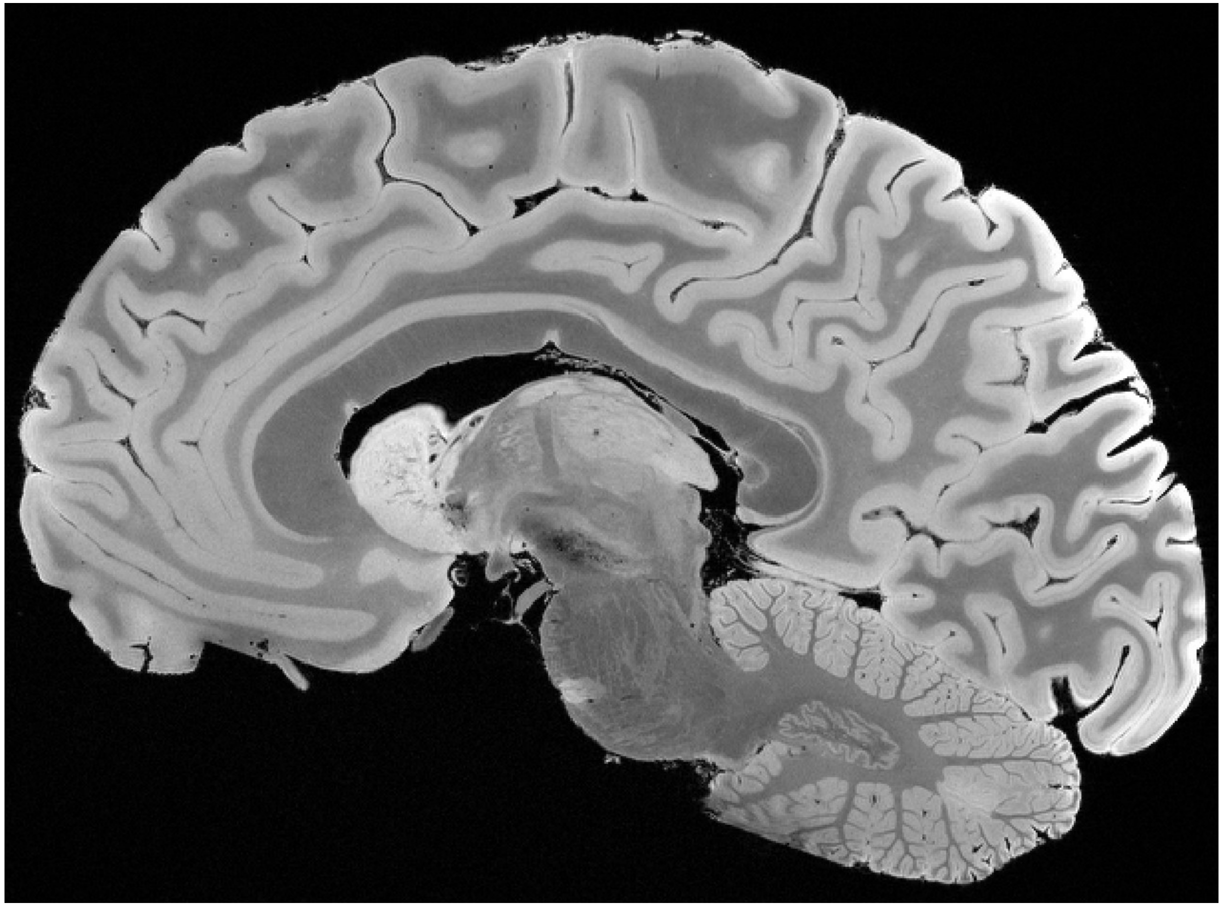

Suppl. Figure S6:

Small appreciation of the imaging capability of the “fast URI-FLASH protocol” with an isotropic 270µm resolution (sagittal and coronal reformation, brain #2).

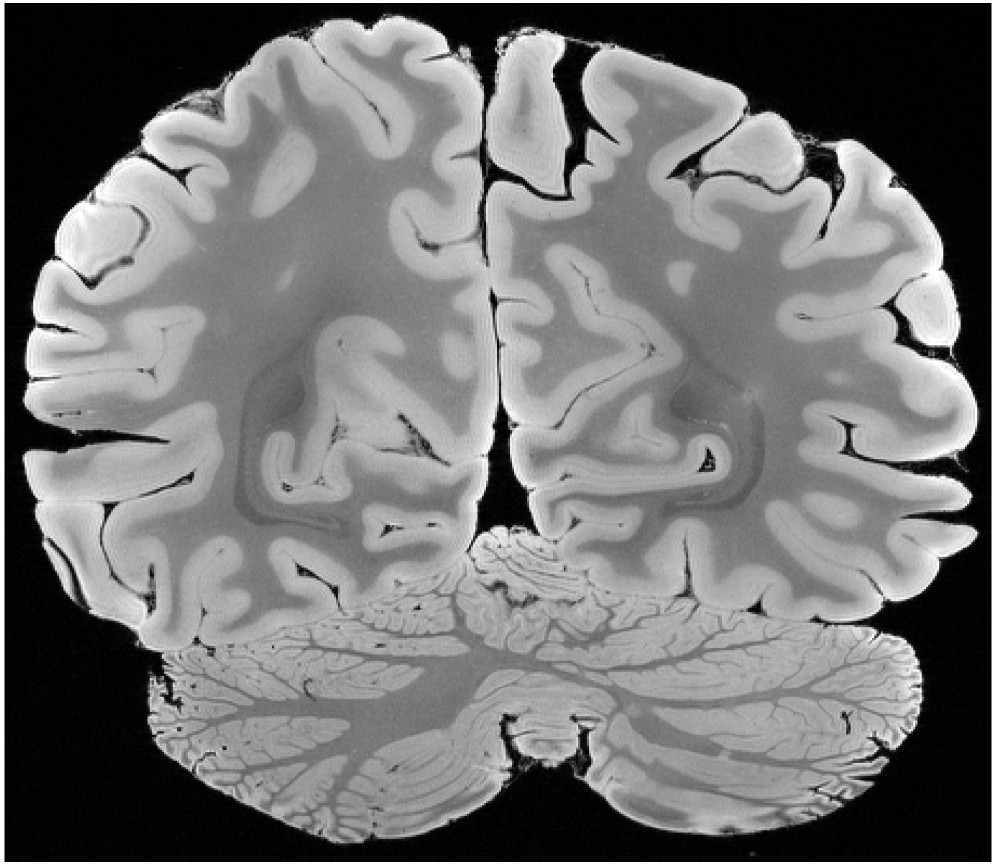

Supplement: Supplementary file 2 — Supplementary Information 2. [file 41598_2021_94891_MOESM2_ESM.pdf]
