# Supplementary material for: Imaging multiple sclerosis pathology at 160 μm isotropic resolution by human whole-brain ex vivo magnetic resonance imaging at 3 T
Source: Sci Rep. 2021 Jul 29;11:15491. doi: 10.1038/s41598-021-94891-1 (PMC8322069; doi:10.1038/s41598-021-94891-1)
Supplement: Supplementary file 3 — Supplementary Information 3. [file 41598_2021_94891_MOESM3_ESM.pdf]

SUPPLEMENTARY INFORMATION: VIDEOS

Imaging Multiple Sclerosis Pathology at 160µm Isotropic Resolution by Human Whole-Brain *Ex Vivo* Magnetic Resonance Imaging at 3T

Weigel, Matthias, PhD [1,2,3,4,*]; Dechent, Peter, PhD [5]; Galbusera, Riccardo, MD [1,2,3]; Bahn, Erik, MD [6]; Nair, Govind, PhD [7]; Lu, Po-Jui, MS [1,2,3]; Kappos, Ludwig, MD [1,2,3]; Brück, Wolfgang, MD [6]; Stadelmann, Christine, MD [6]; Granziera, Cristina, MD, PhD [1,2,3]

[1] Translational Imaging in Neurology (ThINk) Basel, Department of Biomedical Engineering, University Hospital Basel and University of Basel, Gewerbestrasse 14, 4123 Allschwil, Switzerland

[2] Neurologic Clinic and Policlinic, Departments of Medicine, Clinical Research and Biomedical Engineering, University Hospital Basel and University of Basel, Petersgraben 4, 4031 Basel, Switzerland

[3] Research Center for Clinical Neuroimmunology and Neuroscience Basel, University Hospital Basel and University of Basel, Petersgraben 4, 4031 Basel, Switzerland

[4] Division of Radiological Physics, Department of Radiology, University Hospital Basel, Petersgraben 4, 4031 Basel, Switzerland

[5] Department of Cognitive Neurology, MR-Research in Neurosciences, University Medical Center Göttingen, Robert-Koch-Strasse 40, 37075 Göttingen, Germany

[6] Institute of Neuropathology, University Medical Center Göttingen, Robert-Koch-Strasse 40, 37075 Göttingen, Germany

[7] Quantitative MRI core facility, National Institute of Neurological Disorders and Stroke, National Institutes of Health, 10 center drive, Bethesda, Maryland, USA

***Correspondence: Matthias Weigel (matthias.weigel@unibas.ch)**

Materials and Methods

Three videos from the 200µm acquisition of brain #1 were generated, which browse through the entire brain in coronal, sagittal, or transverse direction: After cutting away “empty” image parts in all three directions, the respective 200µm dataset was converted into video streams with 17 frames per second using the software MATLAB RELEASE 2020A (THE MATHWORKS, Natick, MA, USA). The resulting uncompressed *.avi* video files were then encoded each with a H.264 video codec (4000kbps bitrate, software VLC MEDIA PLAYER 3.0.14) to generate compressed *.mp4* video files with reduced file size by a factor of approx. 15.

The videos can also be downloaded from *https://postmortem-mri.matthias-weigel.net*. The file size is between 14MB and 23MB per video.

Video Legends

Video S1: Brain #1, browsing through the 200µm URI-FLASH acquisition slice by slice in coronal slice direction from anterior to posterior.

Video S2: Brain #1, browsing through the 200µm URI-FLASH acquisition slice by slice in sagittal slice direction from right to left.

Video S3: Brain #1, browsing through the 200µm URI-FLASH acquisition slice by slice in transverse slice direction from inferior to superior.
